# Novel CTD tag establishes shark fins as ocean observing platforms

**DOI:** 10.1038/s41598-024-63543-5

**Published:** 2024-06-15

**Authors:** Camille M. L. S. Pagniello, Michael R. Castleton, Aaron B. Carlisle, Taylor K. Chapple, Robert J. Schallert, Michael Fedak, Barbara A. Block

**Affiliations:** 1 Oceans Department, Stanford University, Pacific Grove, 93950 USA; 2 Hawaiʻi Institute of Marine Biology, University of Hawaiʻi at Mānoa, Kaneohe, 96744 USA; 3 School of Marine Science and Policy, University of Delaware, Lewes, 19958 USA; 4 Coastal Oregon Marine Experiment Station, Oregon State University, Newport, 97365 USA; 5 SMRU Instrumentation, Scottish Oceans Institute, St Andrews, KY16 8LB UK

**Keywords:** Marine biology, Physical oceanography, Animal migration, Biooceanography, Physical oceanography

## Abstract

Animal-borne tags are effective instruments for collecting ocean data and can be used to fill spatial gaps in the observing network. We deployed the first conductivity, temperature, and depth (CTD) satellite tags on the dorsal fin of salmon sharks (*Lamna ditropis*) to demonstrate the potential of sharks to monitor essential ocean variables and oceanographic features in the Gulf of Alaska. Over 1360 km and 36 days in the summer of 2015, the salmon shark collected 56 geolocated, temperature-salinity profiles. The shark swam through a plume of anomalously salty water that originated from the “Blob” and encountered several mesoscale eddies, whose subsurface properties were altered by the marine heatwave. We demonstrate that salmon sharks have the potential to serve as submesoscale-resolving oceanographic platforms and substantially increase the spatial coverage of observations in the Gulf of Alaska.

## Introduction

In situ ocean observations are critical for improving our predictions of climate change and understanding of ocean variability. Since the year 2000, more ocean data have been collected than over the previous 100 years^1^. This increase in the number of ocean observations is primarily due to more deployments of profiling floats in the Argo array, which currently operates approximately 4000 floats with global coverage^2^. Despite the collection of over two million vertical temperature-salinity profiles in the past 20 years, there are still spatial gaps in the observing network, particularly in polar and coastal regions^3^. As such, to reconstruct a snapshot of the ocean environment, measurements made by a few, dispersed sensors hundreds of kilometers apart need to be interpolated. With ongoing, rapid changes in ocean conditions, it is essential to increase the spatial coverage of oceanographic sampling, especially in the polar regions such as the Gulf of Alaska (GoA), where the effects of climate change are expected to be more pronounced^4^.

Marine mammals such as elephant seals (*Mirounga angustirostris* and *M. leonina*) equipped with conductivity, temperature, and depth (CTD) tags are currently being used to fill observation gaps in under-sampled coastal and polar regions^3^. Since 2002, over 650,000 temperature-salinity profiles have been collected using animal-borne tags^5^. Most of these observations are in high latitude waters, leading to significant insights about water mass characteristics^6^, frontal location and structure^7,8^ as well as sea-ice formation^7,9,10^ in this dynamic region. Additionally, data collected by CTD tags on elephant seals has the appropriate temporal and spatial resolution to estimate submesoscale processes^11^. These studies and others summarized in McMahon et al.^5^ show that oceanographic data collected by animal-borne tags can be used to enhance the capabilities of global observing networks to monitor essential ocean variables.

Other large, non-air breathing marine animals such as sharks that are commonly equipped with pop-up satellite archival transmitting (PAT) tags that measure depth, temperature and light could also be used as platforms to collect temperature-salinity profiles^12,13^. Here, we utilize the first, custom-built CTD-Satellite Relay Data Logger (SRDL) tag designed for the dorsal fin of a salmon shark (*Lamna ditropis*) to investigate subsurface temperature and salinity anomalies in the GoA (i.e., between 50 to 60 ºN and 150 to 125 ºW) during the “Blob”^14,15^, a large patch of anomalously warm water in the northeast Pacific that appeared in the winter of 2013–2014 and persisted until 2016. Additionally, we utilize “double tagged” salmon sharks that have previously been tagged with both PAT and smart position or temperature (SPOT) transmitting tags in the GoA^16–19^ to evaluate the full potential of shark-collected profiles for resolving submesoscale flows and show the potential spatiotemporal coverage of temperature-salinity profiles in the GoA if all salmon sharks tagged with only SPOT transmitting tags over a 17-year period had been instrumented with robust CTD-SRDL fin tags.

## Results

### CTD-SRDL fin tag design and successful deployment

**Figure 1:**
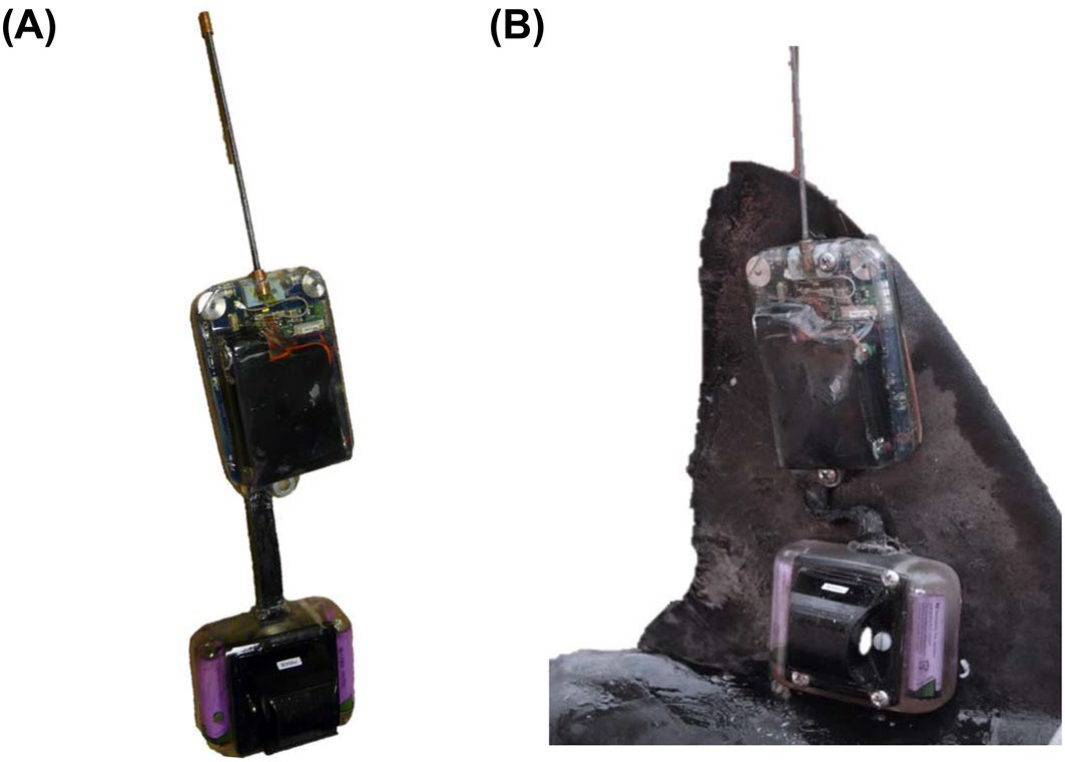
Design of “twin” CTD-SRDL fin tag. (**A**) “Twin” CTD-SRDL fin tag (**B**) deployed on the dorsal fin of a salmon shark. The CTD sensor is located in the package placed closer to the body of the shark while the satellite transmitter and its antenna are in the package placed near the tip of the dorsal fin.

Two distinct designs of the CTD-SRDL fin tag were custom built to mount on the large dorsal fin of a salmon shark. The first design (referred to as “twin”) involved two separate packages, one for the CTD sensor and one for the satellite transmitter and its antenna (Fig. 1A). For a shark to transmit data, the top of its dorsal fin must be out of the water. As such, this design focused on having the satellite transmitter and its antenna as high up as possible on the fin to maximize transmission opportunities. The CTD sensor required immersion to collect temperature, salinity, and depth data and thus, was positioned at the base of the fin, closer to the body of the shark, to ensure that it remained submerged. To enable this separation while also minimizing the size of the tag such that it was small enough to put on a salmon shark’s dorsal fin, the two packages were connected by a ribbon cable. We hypothesize that this ribbon cable, which had limited elasticity and no protection, was a likely source of failure. The second design (referred to as “single”) combined these two components into a single package, with the bulkier components including the CTD sensor at the bottom of the tag (Fig. S1). The downside of this package was its large size. Unfortunately, this tag failed just prior to its deployment on a salmon shark.

**Figure 2:**
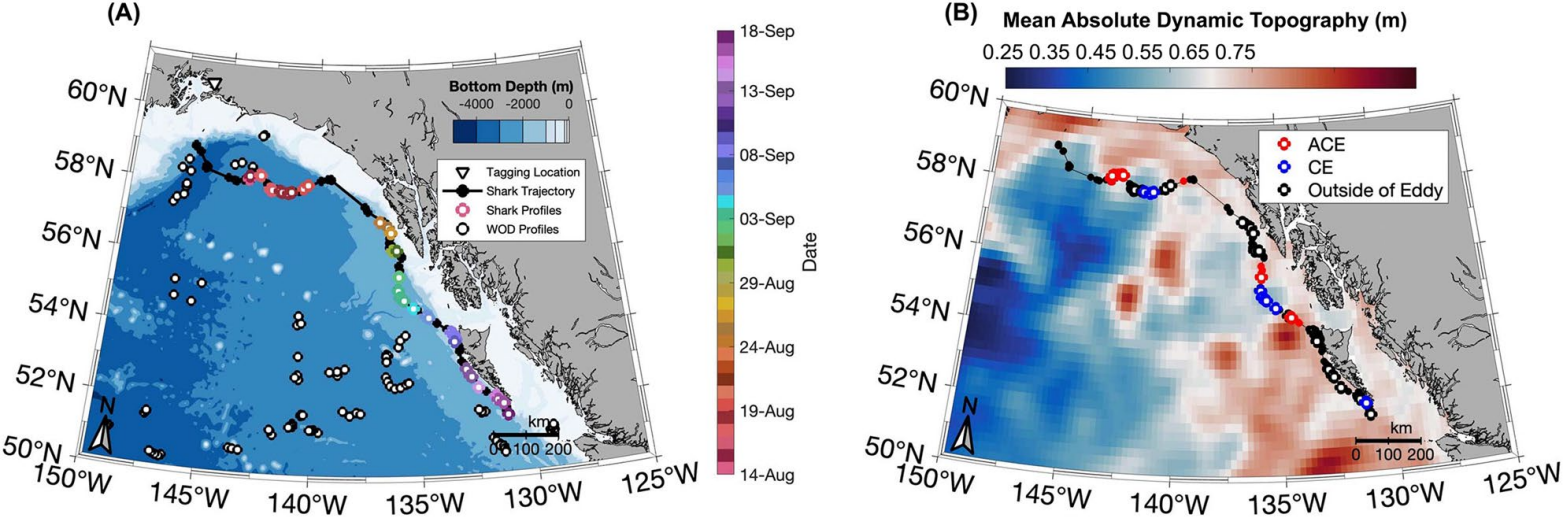
Trajectory of salmon shark equipped with CTD-SRDL fin tag in the Gulf of Alaska between August 14 and September 18, 2015. (**A**) Locations of shark-collected temperature-salinity profiles (circles colored by date) and other ARGOS location estimates (black dots) taken by the tag. Also shown are locations of profiles from the World Ocean Database (black circles) over this period. White triangle with black outline denotes the tagging location. (**B**) Mean absolute dynamic topography over the 36-day CTD-SRDL fin tag deployment with ARGOS location estimates and locations of shark-collected temperature-salinity profiles colored by whether the shark is in an anticyclonic eddy (ACE, red), cyclonic eddy (CE, blue) or outside of eddy (black) is also showed.

**Figure 3:**
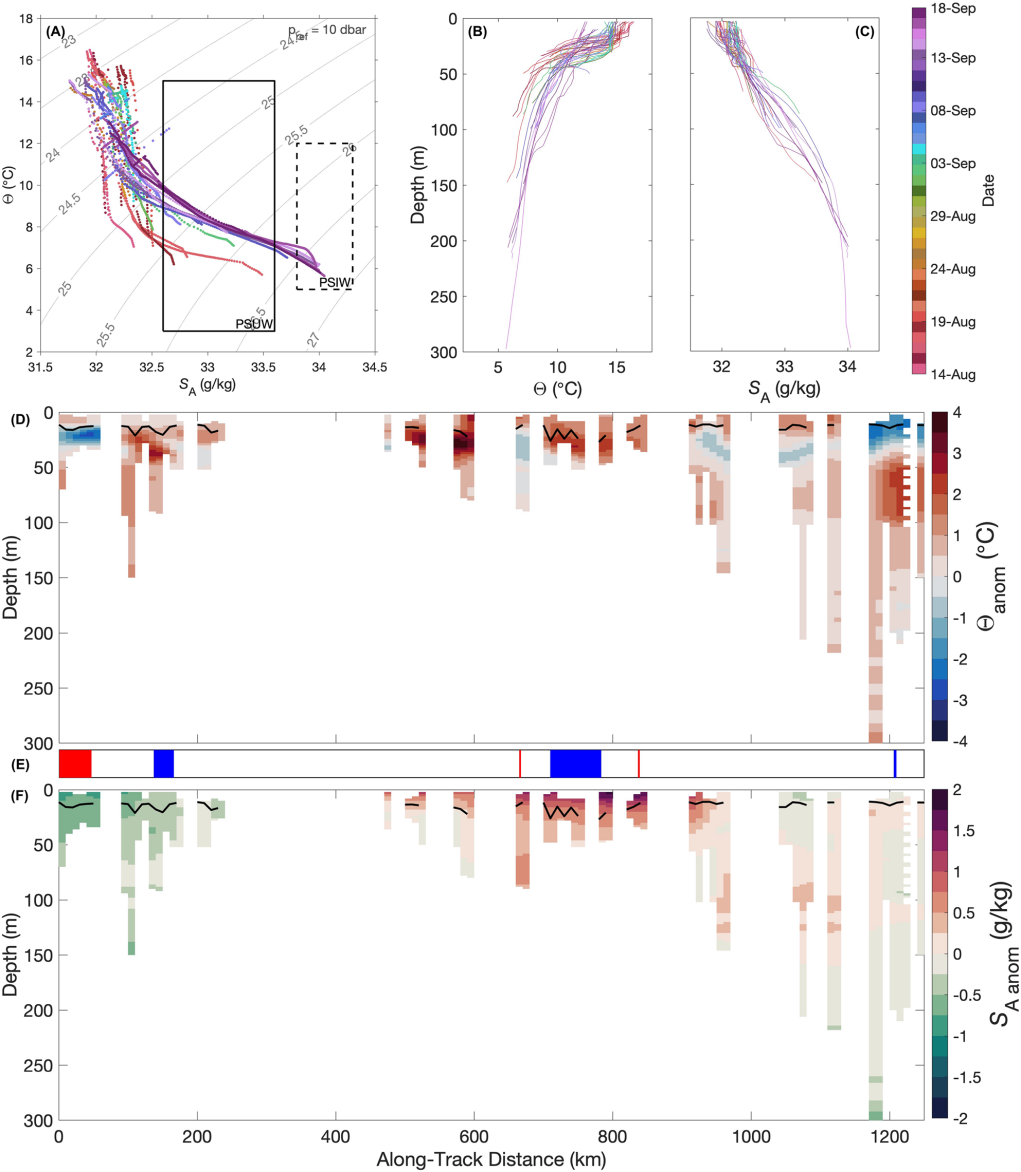
Water properties and anomalies along the trajectory of the salmon shark. (**A**) Temperature-salinity diagram of shark-collected (**B**) conservative temperature (Θ, °C) and (**C**) absolute salinity (*S*_*A*_, g/kg) profiles. Profiles are colored by date. Temperature-salinity characteristics of water masses in the Gulf of Alaska are indicated with boxes: Pacific Subarctic Upper Water (PSUW, 3.0 to 15.0 °C, 32.6 to 33.6 ‰, solid black line) and Pacific Subarctic Intermediate Water (PSIW, 5.0 to 12.0 °C, 33.8 to 34.3 ‰, dashed black line). Depth (m) by along-track distance (km) section of (**D**) conservative temperature and (**F**) absolute salinity anomalies with (**E**) eddy polarity (red = anticyclone, blue = cyclone).

A salmon shark (precaudal length = 157 cm) was equipped with a “twin” design CTD-SRDL fin tag in Port Gravina, Prince William Sound, Alaska during August 2015 (Fig. 1B). The tag successfully transmitted geolocated and time-stamped temperature-salinity profiles through the ARGOS satellite system. From August 14 to September 18, 2015, the shark equipped with this CTD-SRDL fin tag traveled 1360.6 km southeast along the North American continental shelf to Kunghit Island, British Columbia in the Haida Gwaii archipelago (Fig. 2A; Table S2). A total of 56 geolocated, temperature-salinity profiles were collected with maximum depths ranging from 24 to 297 m (µ ± σ: 67 ± 60 m) (Fig. 3B and C). Recorded conservative temperatures (Θ) ranged from 5.6 to 16.5 °C (Fig. 3A). The warmest temperatures (i.e., > 15 °C) were recorded in the top 30 m of the water column in late August 2015 (Fig. S4A). Absolute salinity (*S*_*A*_) ranged from 31.8 to 34.0 g/kg (Fig. 3A).

### Anomalies along the continental margin of the eastern Gulf of Alaska during the “Blob”

Nearly three-quarters of the conservative temperature anomalies measured by the CTD-SRDL fin tag were positive (i.e., warmer) (Fig. 3D). The distribution of Θ anomalies had a mean greater than zero and was negatively skewed, with most anomalies occurring between 0.5 to 1.5 °C (Fig. S5A, Table S3). The strongest Θ anomalies (i.e., greater than 3 °C) were recorded about 20 m below the surface, with the highest recorded off Sitka, AK extending down to 45 m (Fig. 3D). Negative (i.e., colder) anomalies were almost exclusively encountered by the shark between depths of 20 to 45 m and most commonly when the shark was close to the coast. Anomalously cold temperatures were also recorded from the surface down to 35 m near Kunghit Island, BC.

Absolute salinity anomalies measured by the CTD-SRDL fin tag were split evenly between positive (i.e., saltier) and negative (i.e., fresher) anomalies (Fig. 3F). The distribution of *S*_*A*_ anomalies had a mean slightly greater than zero and was positively skewed, with most anomalies occurring between − 0.25 to 0.25 g/kg (Fig. S5B, Table S3). Only salinity profiles at the northern extent of the shark’s trajectory showed strong and consistent negative *S*_*A*_ anomalies throughout the water column (Fig. 3F). When the salmon shark was located near the coast off Sitka, AK, large positive *S*_*A*_ anomalies were recorded at the surface. As the shark moved farther south, these salty anomalies intensified and extended down to 75 m. However, near Kunghit Island, BC, small fresh anomalies occurred haphazardly throughout the water column.

### Mesoscale eddies encountered by the CTD-SRDL fin tagged shark

Along its trajectory, the shark equipped with the CTD-SRDL fin tag encountered seven mesoscale eddies along the continental margin (Fig. 2B, Table S4). Temperature-salinity profiles were collected in three anticyclones and three cyclones. No profiles were collected in the ACE encountered on August 21, 2015. From August 14 to 18, 2015, at the northern extent of its trajectory, the shark transited across an anticyclonic eddy (ACE) that deepened the mixed layer depth (MLD) (Fig. 3D–F). Cold and fresh anomalies only occurred between 15 to 30 m in this eddy, with warm anomalies up to 1 °C extending down from the surface to 15 m (Fig. 3D and F). The shark also encountered a cyclonic eddy (CE) on August 19, 2015, but only remained within its bounds for about 12 h and recorded no cold anomalies (Fig. 2B, Table S4).

The next three eddies through which the shark transited were located south of Sitka, AK (Fig. 2B). Here, the shark spent more time in the CE than in the two ACE it encountered, with few profiles taken within the bounds of the ACEs (Table S4). Waters in these eddies were anomalously salty throughout the water column and warm in the top 25 m (Fig. 3D–F). The shark spent less than 5 h in the final CE it encountered at the southern tip of Kunghit Island, BC (Fig. 2B, Table S4). Small fresh and salty anomalies were measured throughout the water column, with strong cold anomalies in the top 25 m and strong warm anomalies below extending down to 100 m (Fig. 3D and F).

### Comparison to co-located Argo profiling float

**Figure 4:**
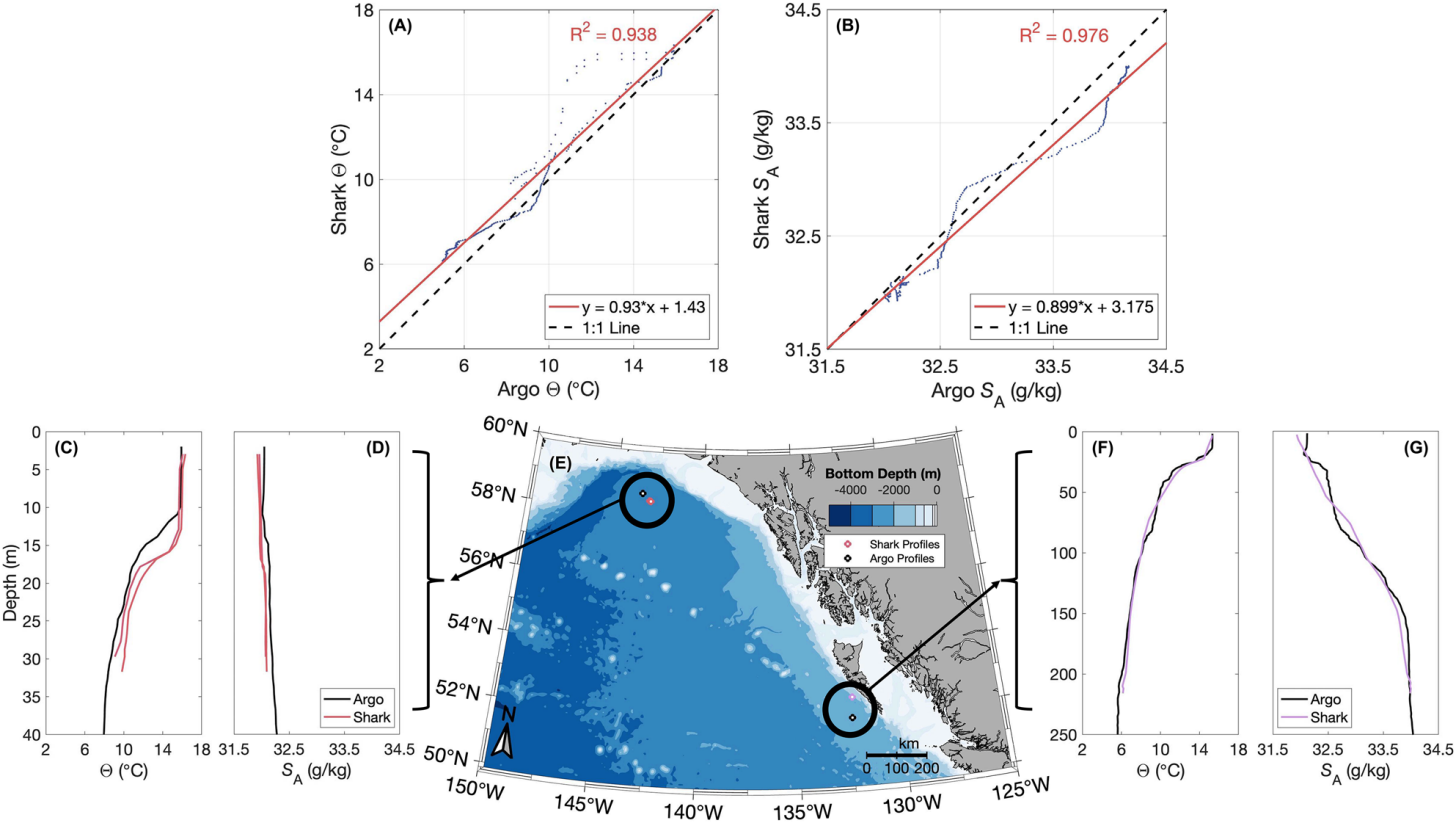
Comparison between shark-collected temperature-salinity profiles and co-located Argo profiling float. Linear regression of shark-collected and Argo profiling float (**A**) conservative temperature (Θ, °C) and (**B**) absolute salinity (*S*_*A*_, g/kg) data co-located in time (± 1 day) and space (± 0.75° latitude and longitude). The locations of co-located shark (pink and purple) and Argo (black) (**C** and **F**) Θ and (**D** and **G**) *S*_*A*_ profiles are shown on (**E**) the map.

Over the 36-day CTD-SRDL fin tag deployment, 112 Argo profiling float and 7 CTD casts were independently collected within the footprint of the shark’s trajectory spanning 150 to 125°W and 50 to 60°N (Fig. 2A). However, only 2 unique Argo profiles and 3 unique shark profiles were co-located within ± 1 day and ± 0.75° latitude and longitude (Fig. 4E). The mean (± standard deviation) of the residuals of the linear regression models fitted to the co-located data was 0.514 ± 0.601 °C and 0.088 ± 0.079 g/kg, respectively (Fig. 4A and B).

Shark-collected profiles at the northern end of the shark’s overall trajectory had a similar shape to their co-located Argo profile but the MLD extended approximately 3 m deeper in the shark-collected profiles (Fig. 4C). In addition, the CTD-SRDL fin tag measured slightly fresher (about 0.2 g/kg) salinities than the Argo profiling float at these locations (Fig. 4F). The shark-collected profile at the southern end of the shark’s overall trajectory was also very similar to its co-located Argo profile in both shape and measured values (Fig. 4D and G).

### Feasibility of salmon shark tag data for resolving submesoscale flows and potential spatiotemporal coverage in the GoA

**Figure 5:**
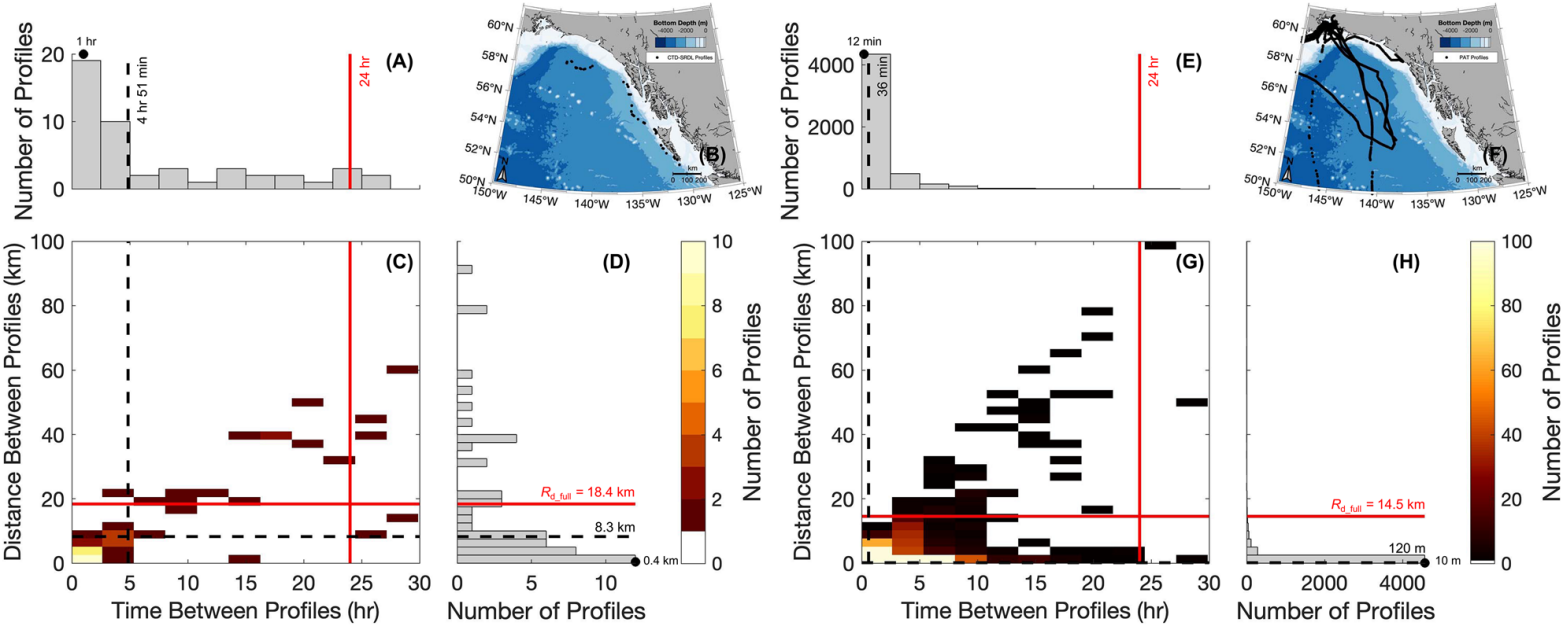
Ability of shark-collected profiles to resolve submesoscale flows. Histograms showing the (**A**) spatial and (**D**) temporal resolution of shark-collected temperature-salinity profiles (black dots) from the CTD-SRDL fin tag in the Gulf of Alaska shown in (**B**). (**C**) A heatmap showing the relationship between distance and temporal resolution for data shown in panels (**A**) and (**D**). (**E**) through (**H**) are the same panels as (**A**) through (**D**) but for temperature-depth profiles from “double tagged” sharks with recovered PAT tags. Color in the heatmap represents the number of profiles. The red lines show the spatial (full-depth Rossby deformation radius; R_d_full_) and temporal cutoff for submesoscale estimates, respectively. The 50th percentile of the data (black dashed lines) and modal value (black dots) are also shown. Data in all panels is cut to 100 km and 30 h.

For the shark CTD-SRDL fin tag, 66.1% of profiles were horizontally separated by less than the mean (± σ) full-depth Rossby radius of deformation (*R*_d_full_) of 17.7 ± 0.7 km (Fig. 5B and C). About 82.1% of shark dives were separated by time intervals less than 24 h (Fig. 5A and B). The modal horizontal spatial and temporal resolution of shark-collected temperature-salinity profiles was 390 m and 1 h, respectively.

Salmon sharks were tagged with PAT and/or SPOT tags between 2002 and 2019 as part of the Tagging of Pacific Pelagics (TOPP) program^20^. “Double tagged” sharks whose PAT tags were recovered (i.e., instances when the tag is physically retrieved and the entire, high resolution archival record from a tag is available) collected a total of 8750 temperature-depth profiles in GoA. The deepest profile spanned 372 m, with 1422 profiles spanning 100 to 200 m, 428 between 200 and 300 m and 36 greater than 300 m (Fig. S9). If only shark-collected temperature profiles in the GoA spanning at least 24 m are considered, 98.6% of profiles were horizontally separated by less than the mean (± σ) *R*_d_full_ of 8.9 ± 5.6 km (Fig. 5E and F). About 99.8% were separated by time intervals less than 24 h (Fig. 5D and E). The modal horizontal spatial resolution of profiles was 10 m and temporal resolution was 12 min, with 50% of profiles separated by less than 120 m and 36 min.

**Figure 6:**
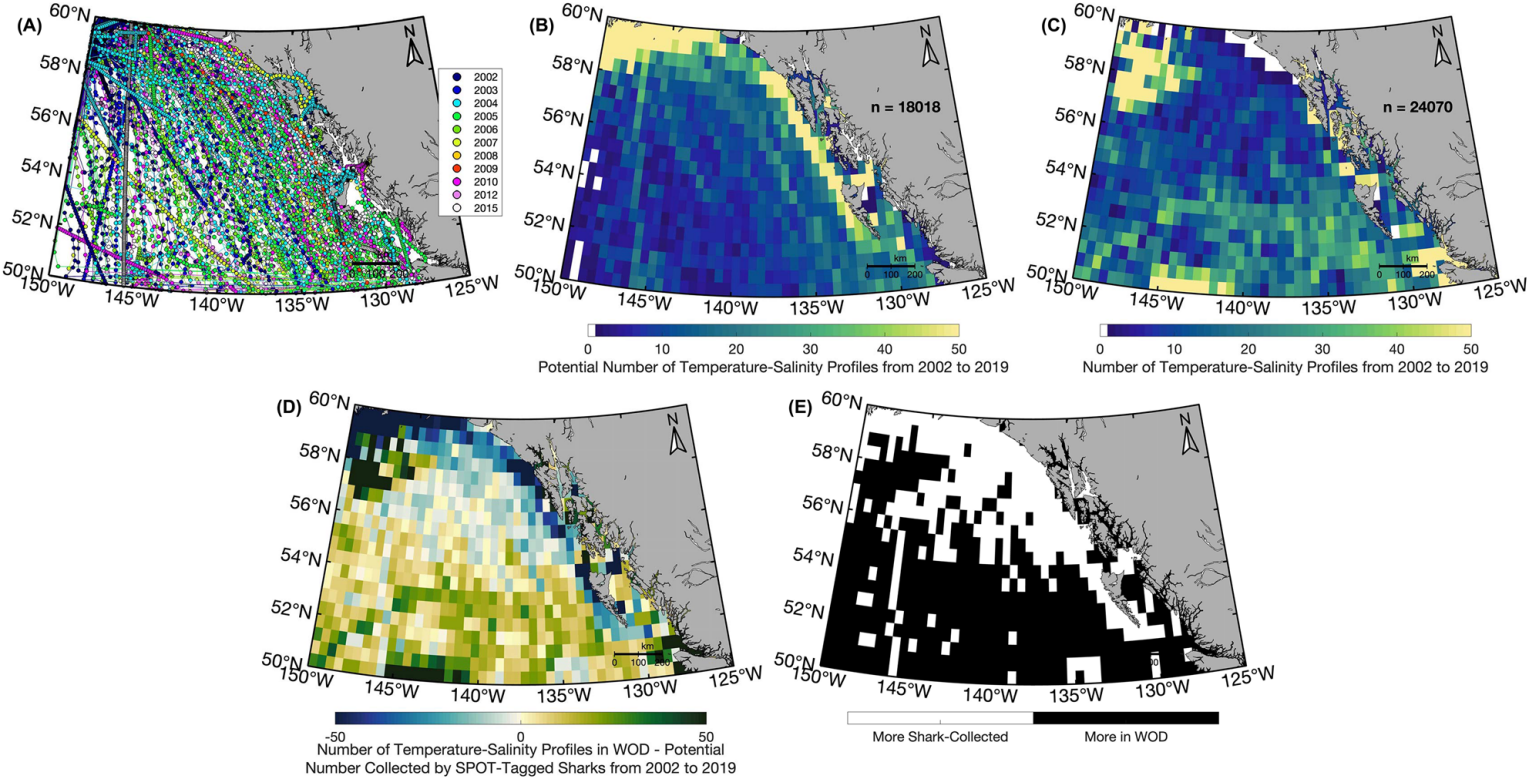
Potential geographic coverage of shark-collected temperature-salinity profiles compared to those in the World Ocean Database between 2002 and 2019 in the Gulf of Alaska. (**A**) Tracks of SPOT-tagged salmon sharks colored by deployment year. Each dot represents a potential profile every 17.2 h. (**B**) Minimum number of temperature-salinity profiles that could have been collected if all SPOT-tagged salmon sharks were instrumented with CTD-SRDL fin tags and (**C**) the number of temperature-salinity profiles in the World Ocean Database (WOD) on days when salmon sharks were present in the Gulf of Alaska. (**D**) Difference between the number of profiles in the WOD and the minimum number of shark-collected profiles, with (**E**) regions where there would be more profiles in the WOD shown in black and more shark-collected profiles in white. Bins are 0.5° latitude and 0.5° longitude.

**Figure 7:**
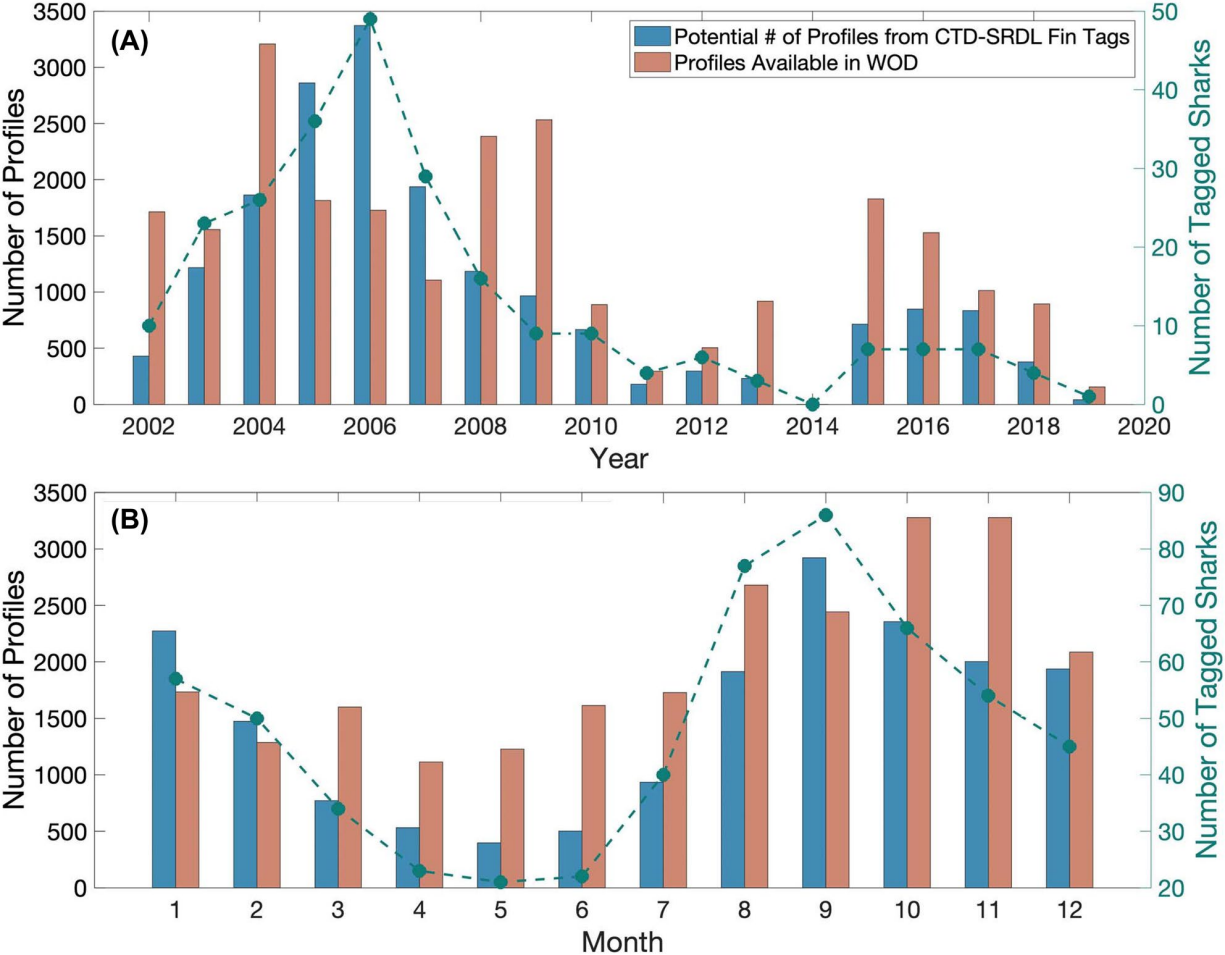
Potential temporal coverage of shark-collected profiles compared to those in World Ocean Database between 2002 and 2019 in the Gulf of Alaska. Minimum number of temperature-salinity profiles that could have been collected if all SPOT-tagged salmon sharks were instrumented with CTD-SRDL fin tags (blue) by (**A**) year and (**B**) month. Also shown are the number of temperature-salinity profiles in the World Ocean Database on days when salmon sharks were present in the Gulf of Alaska (red) as well as the number of SPOT-tagged (dashed green line and circles) sharks.

If all salmon sharks equipped with SPOT tags during the TOPP program had been equipped with CTD-SRDL fin tags, a minimum of 18,018 profiles could have been collected between 2002 and 2019 (Fig. 6A). When the highest number of sharks were SPOT-tagged in the GoA (n = 49) in 2006, 3372 temperature-salinity profiles could have been collected by sharks alone (Fig. 7A). Most profiles would have been collected between August and February (Fig. 7B) (i.e., when salmon sharks spend the most time in the GoA^19^) and would have been concentrated in the northwest corner of the GoA and along the coast (Fig. 6C and D).

## Discussion

### Insights about changes in subsurface properties during the “Blob” in the Gulf of Alaska

This study reports the results from the first successful deployment of a CTD-SRDL tag on the fin of a salmon shark which traveled along the coast of the GoA during the “Blob”. The abundance of warm temperature anomalies measured by the CTD-SRDL fin tag along the shark’s trajectory aligns with other observations that show that the offshore warm waters of the “Blob” became attached to the coast in August 2015^21^. Additionally, temperature profiles collected by the CTD-SRDL fin tag showed that the GoA was overall warmer in August than in September 2015, when the influence of the “Blob” began to slowly dissipate. They also showed that the anomalously warm water of the “Blob” had retreated from the surface and was located between 20 to 45 m. These findings are corroborated by both sea surface temperature maps (Fig. S6A) as well as measurements made in August 2015 along the historical transect line known as Line-P that extends from the mouth of the Juan de Fuca Strait to Ocean Station Papa at 50 ºN and 145 ºW in the GoA (i.e., south of the shark’s trajectory)^21^. Further, cold anomalies recorded from the surface down to 35 m near Kunghit Island, BC can also be seen in satellite imagery taken between September 4 and 18 (Fig. S6B and C). This suggests that this anomalously cold surface water is likely due to runoff from snowmelt, one of the many cold, freshwater inputs into the GoA that begins to peak in September^22,23^.

All *S*_*A*_ measurements made by the shark CTD-SRDL fin tag in the top 50 m of the water column were typical of shelf waters in the GoA (i.e., 31 < *S*_*A*_ < 32.5)^23^ (Fig. S4B). Pacific Subarctic Upper Water and Pacific Subarctic Intermediate Water^24^ were only encountered by the shark at depths between 60 to 150 m and below 175 m, respectively. Previous reports of the “Blob” in the GoA are typically accompanied by observations of unusually fresh conditions^21,25–28^. Waters with fresh anomalies that originated from the west coast of the northern Pacific in December 2010 appeared offshore in the northeast Pacific (40 to 50 ºN, 155 to 140 ºW) in the spring of 2012, but only lasted until the spring of 2014^27,29,30^. However, negative *S*_*A*_ anomalies continued to be present in nearshore waters^31^ as well as in the north and western GoA through 2016^25,28,29^ most likely due to increased melting due to the anomalously warm oceanic and atmospheric temperatures. Yet, negative *S*_*A*_ anomalies only occurred throughout the water column at the northern extent of the shark’s trajectory (Fig. 3F). Sea surface salinity anomaly satellite data from September 2015 show that the shark swam through a plume of anomalously salty water carried by the North Pacific Current and Alaska Current from the offshore warm water pool that now consisted of anomalously salty water (Fig. S7). The paucity of in situ oceanographic measurements along the continental margin in the eastern GoA during this time likely contributes to why we could not find any previous reports of this anomalously salty water plume reaching the coast.

In the GoA, large and long-lived mesoscale eddies are common oceanographic features^32–34^. The first ACE encountered by the shark along its trajectory originated from the Sitka formation region^35^, one of three coastal eddy generation sites in the eastern GoA^22,32–34,36,37^. ACEs in the eastern GoA are typically capped by relatively cold and fresh water in the top 30 m of the water column^37^. Warming observed in the top 15 m of the water column of this ACE is, thus, likely due to the “Blob,” which resulted in anomalies of over 2.5 °C at the surface in the GoA^14^. Additionally, ACEs in the GoA are known to have elevated chlorophyll and more nutrients than the surrounding high-nutrient, low-chlorophyll Gulf waters as they tend to transport coastal water into the Subarctic Gyre^36,38^. This, in turn, enhances zooplankton abundance of both coastal and oceanic species within the bounds of the eddy^39–41^. Thus, the increased biological activity within such eddies could be why the shark appeared to be retentive in this feature, spending over 72 h, collecting 6 temperature-salinity profiles, within its bounds (Table S4). This is also consistent with other recent studies that suggest that ACEs aggregate marine top predators, such as sharks and tunas^42,43^. Similarly to ACEs in the eastern GoA, CEs also typically have a cold fresh cap. However, the signal is much weaker^37^. While the 3 temperature-salinity profiles collected by the shark within the CE predictably showed anomalously fresh water, no cold anomalies were observed (Fig. 3D–F). Again, this pattern of anomalies (i.e., slightly cold anomalies becoming slightly warm anomalies) reflects the influence of the “Blob” not only at the surface, but throughout the water column.

Residuals of the linear regression models fitted to co-located, Argo-collected temperature and salinity data are on the same order as those reported in Holser et al.^31^, which compared 86 seal- and Argo-collected profiles co-located within ± 1 day and ± 0.25° latitude and longitude in the northeast Pacific from 2014 to 2017. Differences in the depth of the MLD and fresher anomalies measured by the CTD-SRDL fin tag are because the shark-collected profiles were taken within an ACE. ACEs deepens the MLD and, in the GoA, trap water from the coast that is fresher than Gulf waters^37^. Other small, observable differences between the profiles were likely due to the use of a broken-stick model to simplify the shark-collected profile down to 16 cut points to enable transmission^44^.

### Sharks as oceanographers

Despite their important role in structuring marine ecosystems^45^, submesoscale flows are often under-sampled due to the lack of adequate observations on time scales of hours to days and length scales of 1 to 10 km. Oceanographic profiles collected by animal-borne tags have enormous potential to be a valuable dataset to fill this gap in the existing ocean observing network. Our findings demonstrate that it is feasible for salmon sharks to serve as submesoscale-resolving oceanographic platforms. We found that the profiles collected by the salmon shark instrumented with the CTD-SRDL fin tag had the minimum temporal and spatial spacing required to resolve submesoscale flows. In fact, sharks have the potential collect a higher percentage of submesoscale, spatially resolving profiles than typically collected with CTD-SRDL tags equipped on elephant seals in Antarctica^11^. Additionally, the modal horizontal spatial and temporal resolution of shark-collected temperature-salinity profiles would be smaller than seal-collected profiles. Though, the *R*_d_full_ in the Gulf of Alaska is slightly larger than in Antarctic waters and the shark CTD-SRDL fin tag collected data for a shorter duration. While all seal dives had the temporal spacing required to resolve submesoscale features, only about 82.1% of shark dives were separated by time intervals less than 24 h (Fig. 5A and B). Due to the small size of the CTD-SRDL fin tag dataset (n = 56), we refrained from computing submesoscale buoyancy fluxes that are typically done with high-resolution, recovered datasets^11,46^. However, the submesoscale spatial and temporal separation of profiles collected by recovered PAT tags further supports our claim that salmon sharks could serve as submesoscale-resolving oceanographic platforms especially considering advances in tag technology since this experimental deployment in 2015 that should allow for more profiles to be collected with submesoscale resolution.

We found that, in general, a single salmon shark equipped with a CTD-SRDL fin tag could collect at minimum 509 temperature-salinity profiles per year primarily in the top 200 m of the water column. Given that multiple profiles could be sent during a single transmission, this is a conservative estimate of the total number of profiles that could have been collected by sharks. However, this conservative estimate is fourteen times the number of profiles that an Argo profiling float collects per year (n ≈ 36). If all salmon sharks equipped with SPOT tags between 2002 to 2019 had been equipped with CTD-SRDL fin tags, nearly double the number of temperature-salinity profiles could have been collected in the GoA as only 24,159 profiles from five different types of platforms were deposited in the World Ocean Database (WOD) over this 17-year period (Fig. 6B)^47^. In this case, sharks would have collected twice as many profiles than gliders (n = 8791) or Argo profiling floats (n = 8229) (Fig. S11C and E). Further, 51% more temperature-salinity profiles could have been collected by sharks in 2006 (i.e., when the highest number of sharks were SPOT-tagged) than what is available in the WOD on days when salmon sharks were present in the GoA (Fig. 7A). The potential 3372 shark-collected profiles is also more than what is available in the WOD in any year between 2002 to 2019. However, for this to be operationalized, it would require the development of more robust CTD-SRDL fin tags with deployment durations longer than one month.

This study demonstrates the high potential for salmon sharks to be one of the best oceanographic platforms available in the GoA that can help fill gaps in the current ocean observing network, particularly along the coast. Profiles in the WOD were concentrated in the northeast corner and at the southern end of the GoA while shark-collected profiles would have been concentrated in the northwest corner of the GoA and along the coast. Thus, despite their coastal bias, sharks would be a complimentary ocean observing platform to those included in the WOD that would substantially increase the number of profiles in areas of the GoA where fewer have historically been collected. Salmon sharks equipped with CTD-SRDL fin tags have the potential to sample the water column most similarly to underwater gliders, which typically reach their maximum depth in 1.5 h and cover a horizontal distance of 1.5 km in that time^48^. Though, gliders are typically programmed to profile to 500 m, a depth that only two shark-collected profiles reached in the GoA potentially due to their high use of the continental shelf. However, the extended range of salmon sharks beyond the GoA to the Subtropical Gyre and California Current further increases their utility as a platform^17,19^. Widely investing in the concept of animals as oceanographers beyond this species to other large marine top predators that dive to mesopelagic depths (i.e., greater than 200 m) would enable sampling of a variety of habitats on a global scale and provide new insights into oceanographic changes at a submesoscale resolution.

## Methods

### Ethics statement

This research was conducted in accordance with Stanford University Institutional Animal Care and Use Committee protocols to BAB. Experimental procedures were approved by the Stanford University Administrative Panel on Laboratory Animal Care (APLAC) under protocol APLAC-10765. Additionally, all electronic tagging on salmon sharks was permitted by the Alaska Department of Fish and Game.

### Shark CTD-SRDL fin tag on-board data processing and deployment

The package of the CTD-SRDL fin tag (Sea Mammal Research Unit, St. Andrews, UK) was modified from previous versions built for air breathing marine mammals^49^. The on-board software routines described in Fedak et al*.*^50^ and Photopoulou et al.^51^ were modified to better capture the diving patterns of salmon sharks, accounting for the differences between a shark’s vertical diving behavior and that of air-breathing marine mammals. Instead of creating temperature-salinity profiles based on discrete dives, profiles were constructed when the depth time series extended within 10 m of the surface and was deeper than 30 m. Each profile contains 16 depth points consisting of the minimum depth, maximum depth, 6 broken-stick points, and 8 fixed points, which are set depending on the maximum depth of each dive, to enable its transmission via the ARGOS satellite system (Table S1).

The CTD-SRDL fin tag was deployed on the dorsal fin of an adult female salmon shark in Port Gravina, Prince William Sound, Alaska (60.76855°N, 146.0544°W) in August 2015 (Table S2). To tag the salmon shark, it was lifted out of the water on a custom-built cradle and held upright with two straps that were attached to the cradle. This stabilized the shark, allowing it to lay flat with its eyes covered and gills irrigated with a hose once it was aboard the vessel. To position the tag correctly on the dorsal fin, a template was used to create holes through the dorsal fin with a power drill and custom drill bit. The drill bit was cleaned in alcohol and betadine prior to use. Stainless steel bolts were placed through the CTD-SRDL fin tag package and fin to secure the tag in place. The tag transmitted 71 temperature-salinity between August 7 and September 18, 2015. However, only 56 profiles were associated with a latitude and longitude, and thus, used in the analyses.

### CTD post-processing

A median filter was applied to in situ temperature (°C) and practical salinity (PSU) shark-collected profiles with a window length of 15% of the maximum depth of the profile. Before applying the filtering, profiles were vertically interpolated onto a regular grid of 1-m resolution and detrended to account for the large variability of these ocean properties with depth. We could not correct these measurements for a thermal mass effect as done in Siegelman et al.^52^ because only compressed, low-resolution profiles were available and thus, the sample time interval (Δ_t_) for the transmitted data is unknown. In situ temperature and practical salinity were converted to conservative temperature (Θ, °C) and absolute salinity (*S*_*A*_, g/kg), respectively, using the TEOS-10 equation of state^53^. Following the methodology outlined in Barker and McDougall^54^, density-inversions were removed by adjusting only *S*_*A*_ and keeping Θ unchanged. The resulting profiles never exceed a minimum *N*^2^ threshold, where *N*^2^ is the Brunt-Vaisala frequency (radians^2^/s^2^). The N^2^ threshold was set to 1 × 10^–9^ s^−2^. Profiles with sharp and localized jumps induced by the removal of density-inversions from *S*_*A*_ profiles were omitted from the analysis (n = 1). The mixed layer depth (MLD; m) was estimated using the threshold definition from de Boyer Montégut et al*.*^55^ of Δ*ρ* = 0.03 kg/m^3^ greater than the density at 10 m depth.

### Objective mapping

Discrete Θ and *S*_*A*_ data from shark-collected profiles were objectively mapped individually onto a regular grid (1250 km by 300 m) using a Gaussian autocovariance. Levels were separated by 0.4 km in the horizontal direction, the modal separation of profiles along the shark’s trajectory, and by 2 m in the vertical direction, the modal separation of depths in the 16 cut point profiles. The ratio of noise to signal variance was taken to be 0.1 (or 10%) and the autocorrelation of both *S*_*A*_ and Θ was calculated as a function of lag in space to determine the appropriate length scales in the along-track and depth dimensions for the Gaussian function. The resulting objective map is a minimum mean-square error estimate of a continuous function of either *S*_*A*_ or Θ. We assumed that statistics were stationary and homogeneous. All subsequent calculations use the objectively mapped data, with points with error-to-signal variance greater than 0.4 masked (Fig. S3).

### Anomalies

To compute Θ and *S*_*A*_ anomalies, shark-collected profiles were vertically averaged to match the standard depth levels (i.e., 5 m bins from 0 to 100 m, 25 m from 100 to 500 m, and 50 m bins below 500 m) of 1981–2010 monthly climatologies from the World Ocean Atlas (WOA) 2018^56,57^. The nearest (in both space and time) objectively interpolated mean field on a 1/4º grid was subtracted from shark-collected profile (e.g., Θ_anom_ = Θ_shark_ − Θ_climatology_). Anomalies more than ± 4 standard deviations away from the overall mean were deemed outliers and removed (4.5% and 0% of 619 *S*_*A*_ and Θ measurements, respectively). Descriptive statistics (i.e., mean, standard deviation, skewness and kurtosis) of the distributions of Θ and *S*_*A*_ anomalies were computed.

### Sea surface temperature anomaly data

Fourteen-day composites of sea surface temperature (SST) anomaly data between August 14 to September 18, 2015 were obtained from NOAA CoastalWatch via ERDDAP. Anomaly data were generated by subtracting the Casey and Cornillon climatology^58^ from NOAA’s Advanced Very High Resolution Radiometer (AVHRR) Recorder Global Area Coverage (GAC) SST dataset and mapped onto a 0.1° × 0.1° grid using a simple arithmetic mean.

### Sea surface salinity anomaly data

Monthly sea surface salinity (SSS) anomaly data were obtained from the University of Hawaii’s International Pacific Research Center for the months of August and September 2015 (http://iprc.soest.hawaii.edu/users/oleg/oisss/GLB/Aquarius_SMAP_OISSS_monthly/). Anomaly data were generated by subtracting the optimum interpolation (OI) SSS product-based monthly climatology from the multi-mission OI SSS global monthly dataset, a Level 4 product that combines observations from Aquarius/SAC-D, Soil Moisture Active Passive and Soil Moisture and Ocean Salinity satellite missions into a continuous and consistent SSS data record on a 0.25-degree spatial and monthly temporal grid^59^.

### Eddies and other satellite oceanographic data

Effective contours and centers of eddies in the Gulf of Alaska between August 14 to September 18, 2015, were acquired from the Mesoscale Eddy Trajectory Atlas (META3.2 DT allsat, 10.24400/527896/a01-2022.005.220209) distributed by AVISO+. This delayed-time product uses gridded, global absolute dynamic topography (ADT) to track eddies using the algorithm described in Mason et al.^60^ and Pegliasco et al.^61^.

Daily absolute dynamic topography in addition to the geostrophic velocity anomalies (*u*ʹ and *v*ʹ) over the same area and dates were obtained from a global, delayed-time Level 4 altimetry product (SEALEVEL_GLO_PHY_L4_MY_008_047, 10.48670/moi-00148) that combines data from all available altimeter missions distributed by Copernicus Marine Service. The spatial resolution of the fields is 0.25° × 0.25°. Maps of eddy kinetic energy were created from these products such that: $EKE=\frac{1}{2}({u}^{{\prime}2}+{v}^{{\prime}2})$.

### Oceanographic profiles from the World Ocean Database (WOD)

Profiles collected in the GoA between 50 to 60°N and 150 to 125°W were acquired from the WOD 2018^47^. Between July 16, 2002, and February 17, 2019, 8229 Argo profiling float, 6251 ship-based CTD, 8791 glider, 165 ocean station data and 723 pinniped-collected temperature-salinity profiles were collected on the 3394 days when salmon sharks were present in the GoA. Only the location of profiles with both temperature and salinity data were used in the analyses.

### Argo comparison

Unfortunately, no ship-based CTD measurements were available to compare to the CTD-SRDL fin tag data. As such, we could not apply Roquet et al.’s^62^ delayed-mode calibration methods. To validate the observations, shark-collected profiles were compared to spatially (± 0.75° latitude and longitude) and temporally (± 1 day) co-located CTD and Argo profiling float casts from the WOD^47^. Linear regression models were fit to Θ and *S*_*A*_, respectively.

### Other tag deployments on salmon sharks

As part of the TOPP program^20^, 89 salmon sharks were tagged with both pop-up satellite archival transmitting (PAT) tags (PAT1, 2, 3, 4, Mk10, Wildlife Computers, Inc., WA, USA) and a smart position or temperature (SPOT) transmitting tags (SPOT1, 2, 3, 4, 5, Wildlife Computers, Inc., WA, USA) in July and August of 2002 to 2007 using the techniques described in Weng et al*.*^16^, Weng et al*.*^19^, Carlisle et al*.*^18^ and Coffey et al*.*^17^. By tagging sharks with both PAT and SPOT tags, both temperature-depth data and ARGOS locations could be collected simultaneously. Twenty-seven salmon sharks were tagged with only SPOT tags that recorded ARGOS locations during this period. Forty-four PAT tags deployed on “double tagged” sharks successfully transmitted data (Table S5) and eleven PAT tags were recovered, enabling the retrieval of the full archival record of pressure, temperature, and light (Table S6). However, the SPOT tag associated with one of the recovered PAT tags only provided a few locations on the day of tagging and thus, PAT tag #1704016 was omitted from the analyses. Time series from recovered PAT tags were cropped to match pop-up dates.

In 2008, 2010, 2012 and 2015, an additional 21 salmon sharks were tagged with only SPOT tags (Table S5). Of the 137 SPOT tags deployed between 2002 and 2015, 126 transmitted location data through the ARGOS satellite system. A state space model was fitted to the ARGOS tracks as a quality control step to regularize shark location estimates through time and account for observation error^63^. Tracks from SPOT tags were manually reviewed and cropped to remove poor-quality sections. This included removing erroneous start and end locations, locations on land as well as portions of the tracks where consecutive location estimates were separated by the same distance, leaving 123 SPOT tags to be included in the analyses (Table S5). To estimate the potential of salmon sharks to serve as an oceanographic platform, tracks from these SPOT tags were interpolated to estimate the location of the shark every 17.2 h, the number of estimated hours needed for a single transmission^13^.

### Depth correction, dive detection and profile extraction from recovered PAT tags

A zero offset correction was applied to the depth data of the recovered PAT tags using a custom-written implementation of Luque and Fried's^64^ approach in MATLAB. A window length of 4 min was used for the first filter, a median smoothing step to remove noise from depth data. The second filter was a moving quantile filter with a window length of 30 days and quantile of 0.01.

The start and end of dives within the corrected depth time series were detected based on the prominence of local extremum (i.e., greater than 15 m for local maximums and 5 m for local minimums) and maximum depth during a 4-h window. Local maximums (minimums) were only kept if they were deeper (shallower) than 25% of the maximum depth. Adjacent local maxes (mins) were removed. Each dive contained two profiles (i.e., a downcast and upcast) and each profile had one local maximum and minimum. Both the downcast and upcast were used in the analyses. Depth and temperature data were extracted from the time series, bin averaged, vertically interpolated, and smoothed onto a regular grid of 1-m resolution to create a temperature-depth profile and the location of the profile was estimated by linearly interpolating daily regularized shark locations obtained by fitting a state space model to the ARGOS track. Only profiles that spanned at least 24 m (i.e., the smallest maximum depth of temperature-salinity profiles collected by the CTD-SRDL fin tag) were used in the analyses.

### Profiles of depth and temperature (PDTs)

PAT tags transmit profiles of depth and temperature (PDTs), which record the minimum and maximum temperature at 8 depths between the minimum and maximum depth occupied by the tagged shark in the water column over a 6, 12, or 24-h time interval (Table S5). The midpoint profile is representative of average temperatures and can be used to estimate ocean heat content (OHC)^65^. Temperature-depth profiles were vertically interpolated onto a regular grid of 1-m resolution and the location of the profile was estimated by linearly interpolating the ARGOS track. Only PDTs whose maximum depth was greater or equal to 24 m were used in the analyses.

### Dive spacing resolution

We calculated the distance and time between subsequent profiles in both CTD-SRDL fin tag and recovered PAT tags to quantify the dive spacing resolution. To determine whether the shark’s dive spacing would resolve submesoscale oceanographic features, we estimated the full-depth Rossby radius of deformation (*R*_d_full_; m) along the shark’s trajectory. For a baroclinic ocean, *R*_d_full_ is defined as:

$${R}_{d\_full }= \frac{c}{f},$$

where *c* is the baroclinic gravity-wave phase speed (m/s) and *f* is the Coriolis parameter (s^−1^)^66^.

### Supplementary Information

Supplementary Information.

Supplementary Table S5.

## Data Availability

First-baroclinic gravity wave speeds used to compute the full-depth Rossby deformation radius were provided by E. Oliver (https://ecjoliver.weebly.com/rossby-radius.html). The altimetric Mesoscale Eddy Trajectory Atlas product [META3.2 DT allsat, 10.24400/527896/a01-2022.005.220209; (Pegliasco et al.^61^)] was produced by SSALTO/DUACS and distributed by AVISO+ (https://www.aviso.altimetry.fr/) with support from CNES, in collaboration with IMEDEA. This atlas was downloaded the September 27, 2022 and covers the period from January 1993 to September 2022. Ancillary oceanographic profiles are available from the World Ocean Database 2018: https://www.ncei.noaa.gov/products/world-ocean-database. 1981–2010 monthly climatologies are available from the World Ocean Atlas 2018: https://www.ncei.noaa.gov/products/world-ocean-atlas. All other data used in this study is available upon request to the corresponding author.

## Code availability

All code is available on GitHub: https://github.com/cpagniel/SalmonSharkCTD.
