# Supplementary material for: Novel CTD tag establishes shark fins as ocean observing platforms
Source: Sci Rep. 2024 Jun 15;14:13837. doi: 10.1038/s41598-024-63543-5 (PMC11180183; doi:10.1038/s41598-024-63543-5)
Supplement: Supplementary file 1 — Supplementary Information. [file 41598_2024_63543_MOESM1_ESM.docx]

Supplementary Information for

**Novel CTD fin tag highlights utility of sharks as ocean observing platforms**

Camille M.L.S. Pagniello*, Michael R. Castleton, Aaron B. Carlisle, Taylor K. Chapple, Robert J. Schallert, Michael Fedak and Barbara A. Block

Corresponding author: [cpagniel@stanford.edu](mailto:cpagniel@stanford.edu)

Supplementary Figures and Tables


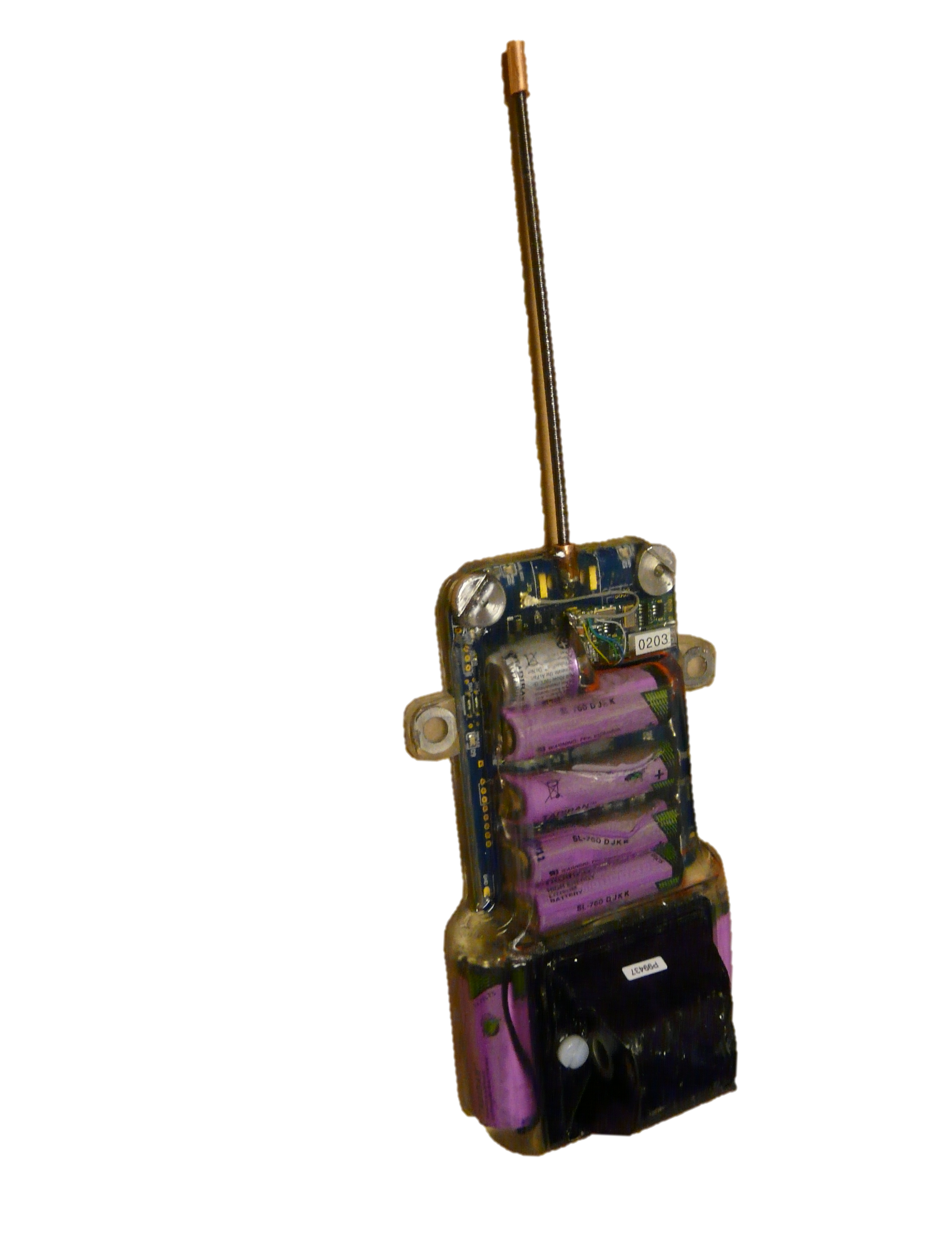


Fig. S1.

Design of “single” CTD-SRDL fin tag.


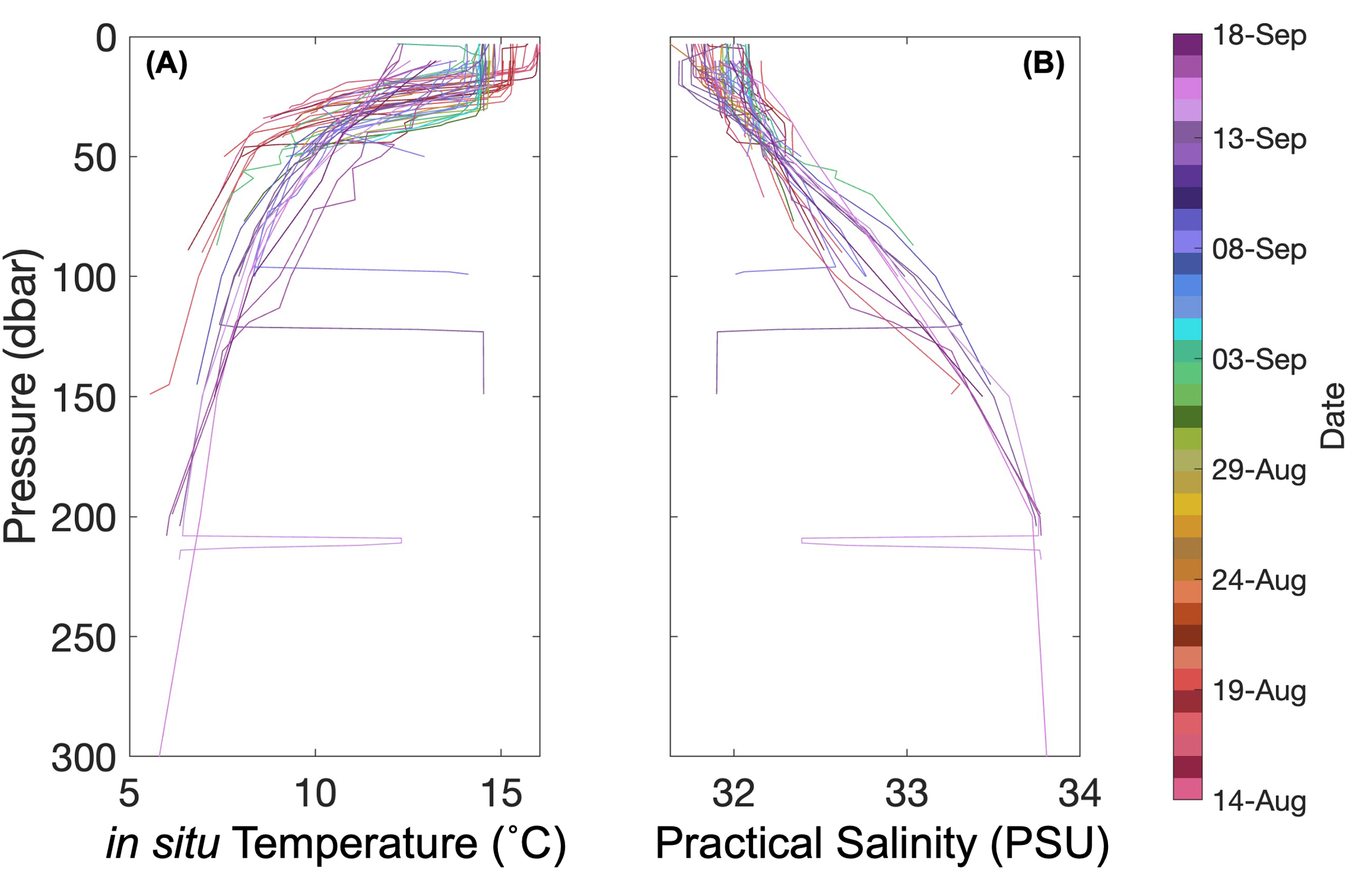


Fig. S2.

(A) *In situ* temperature (ºC) and (B) practical salinity (PSU) shark-collected profiles before post-processing. Profiles are colored by date.


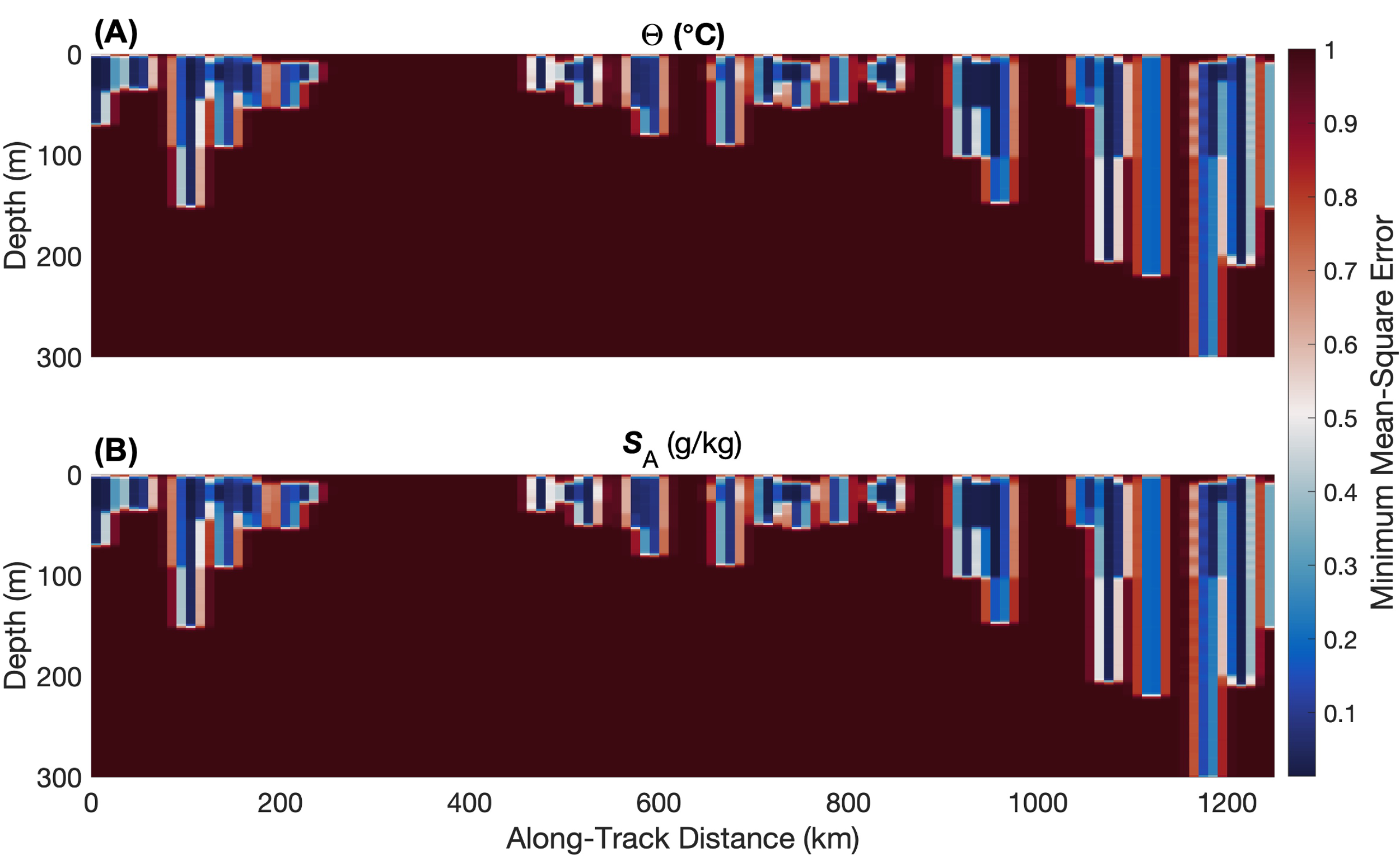


Fig. S3.

Minimum mean-square error estimate of a continuous function of (A) conservative temperature (Θ, ˚C) and (B) absolute salinity (*S_A_,* g/kg) as a function of depth (m) and along-track distance (km).


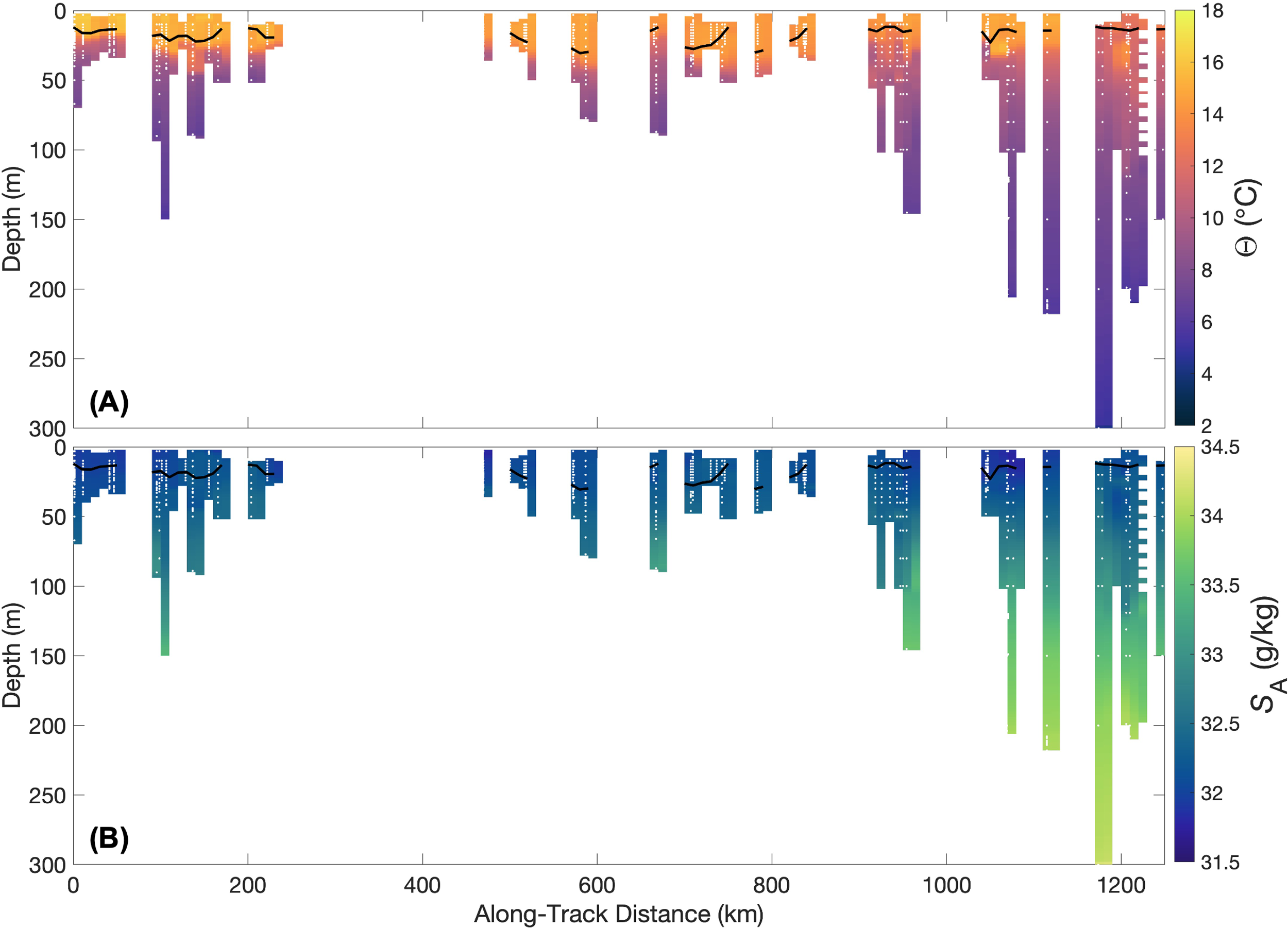


Fig. S4.

Depth (m) by along-track distance (km) section of (A) conservative temperature (Θ, ºC) and (B) absolute salinity (*S_A_*, g/kg) collected by the CTD-SRDL fin tag in the Gulf of Alaska between August 14 and September 18, 2015. Black solid line shows the mixed layer depth (m). White dots show depths of the 16 cut point profiles before CTD post-processing.


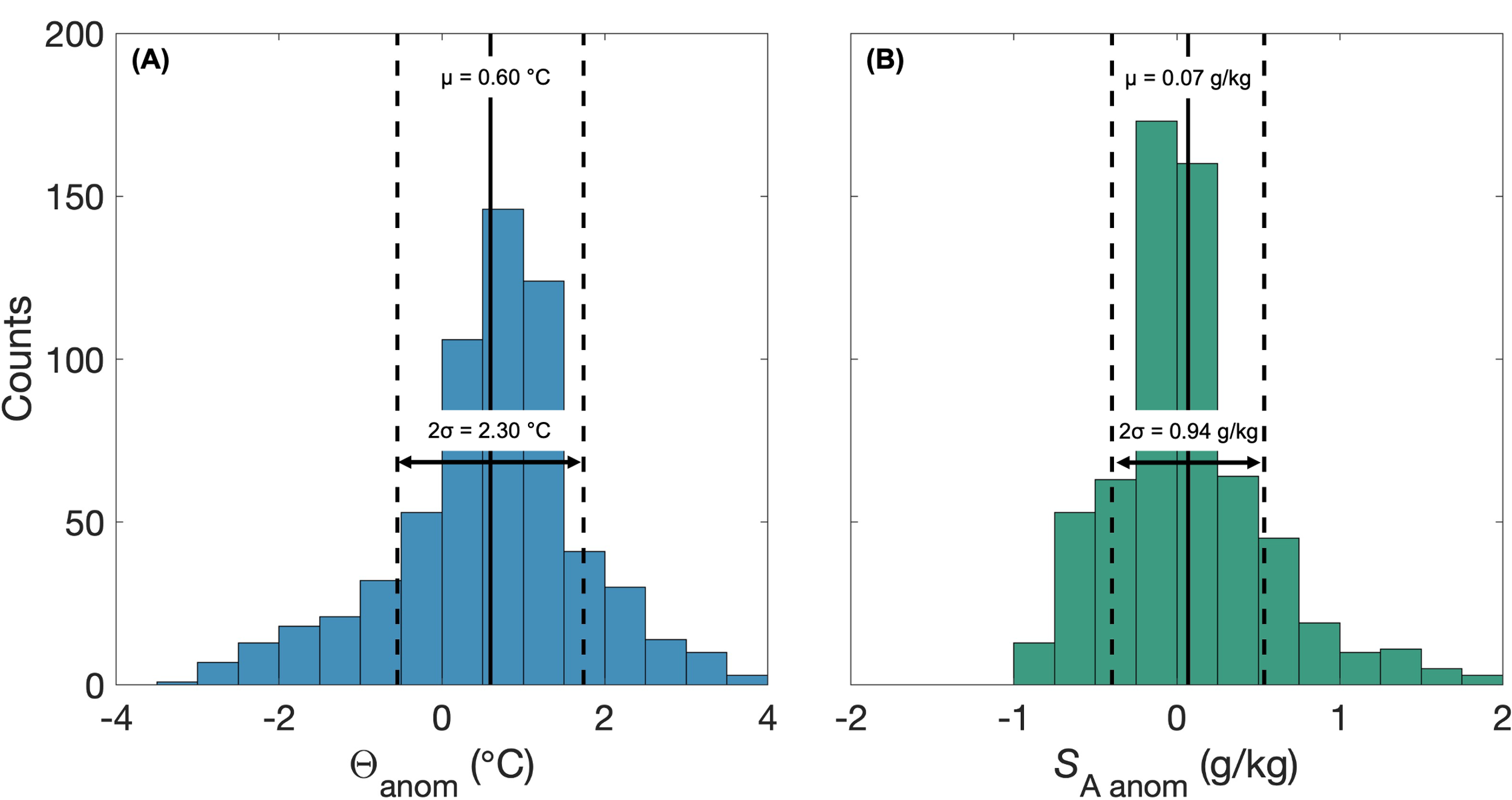


Fig. S5.

Distributions of (A) conservative temperature (Θ, ºC) and (B) absolute salinity (*S_a_*, g/kg) anomalies. Mean (μ; black solid line) and standard deviation (σ; blacked dashed line) are shown.


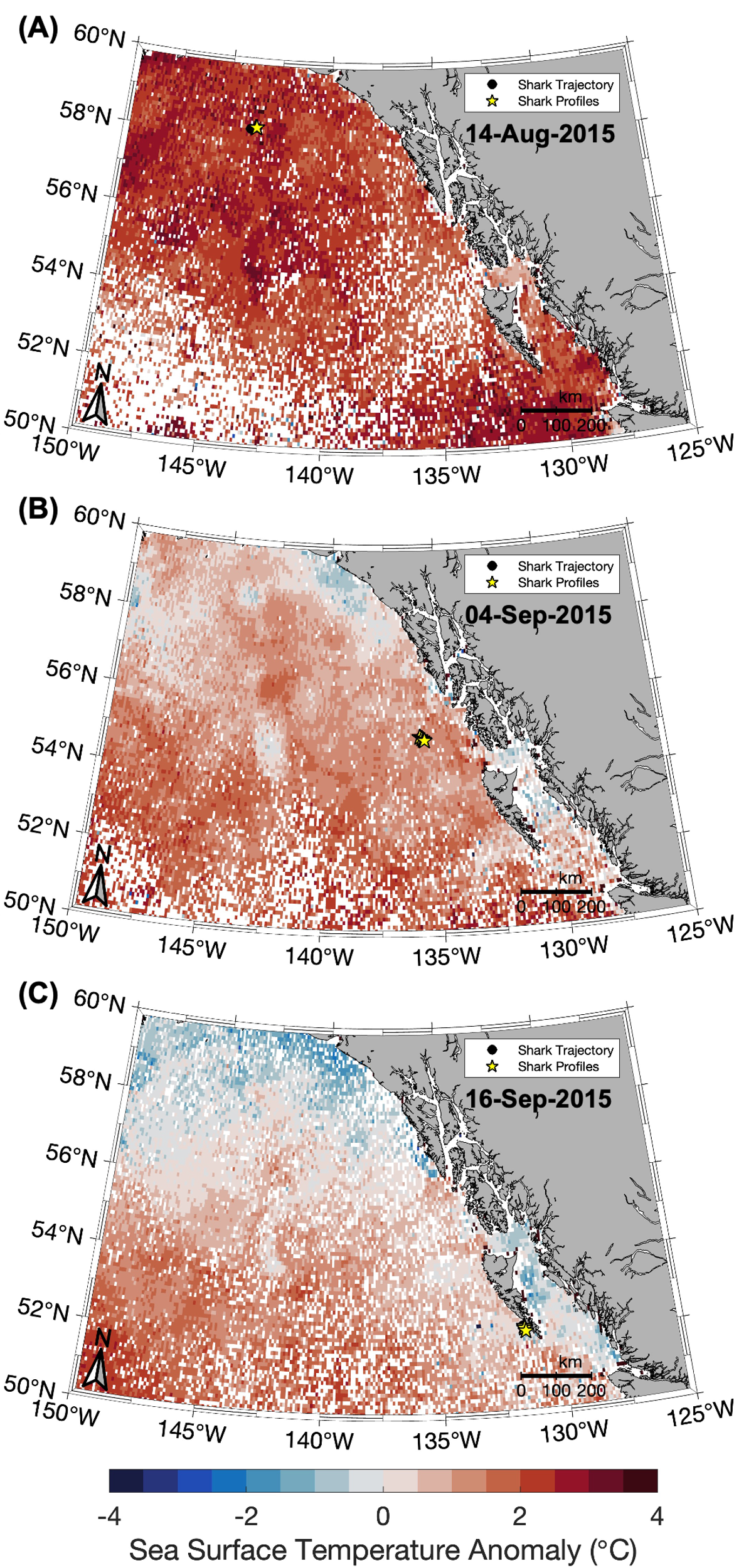


Fig. S6.

Fourteen-day composite sea surface temperature anomaly on (A) August 14, (B) September 4, and (C) September 16, 2015, with ARGOS location estimates (black dots) and locations of temperature-salinity profiles (yellow stars) from CTD-SRDL fin tag showing the dissipation of the “Blob”.


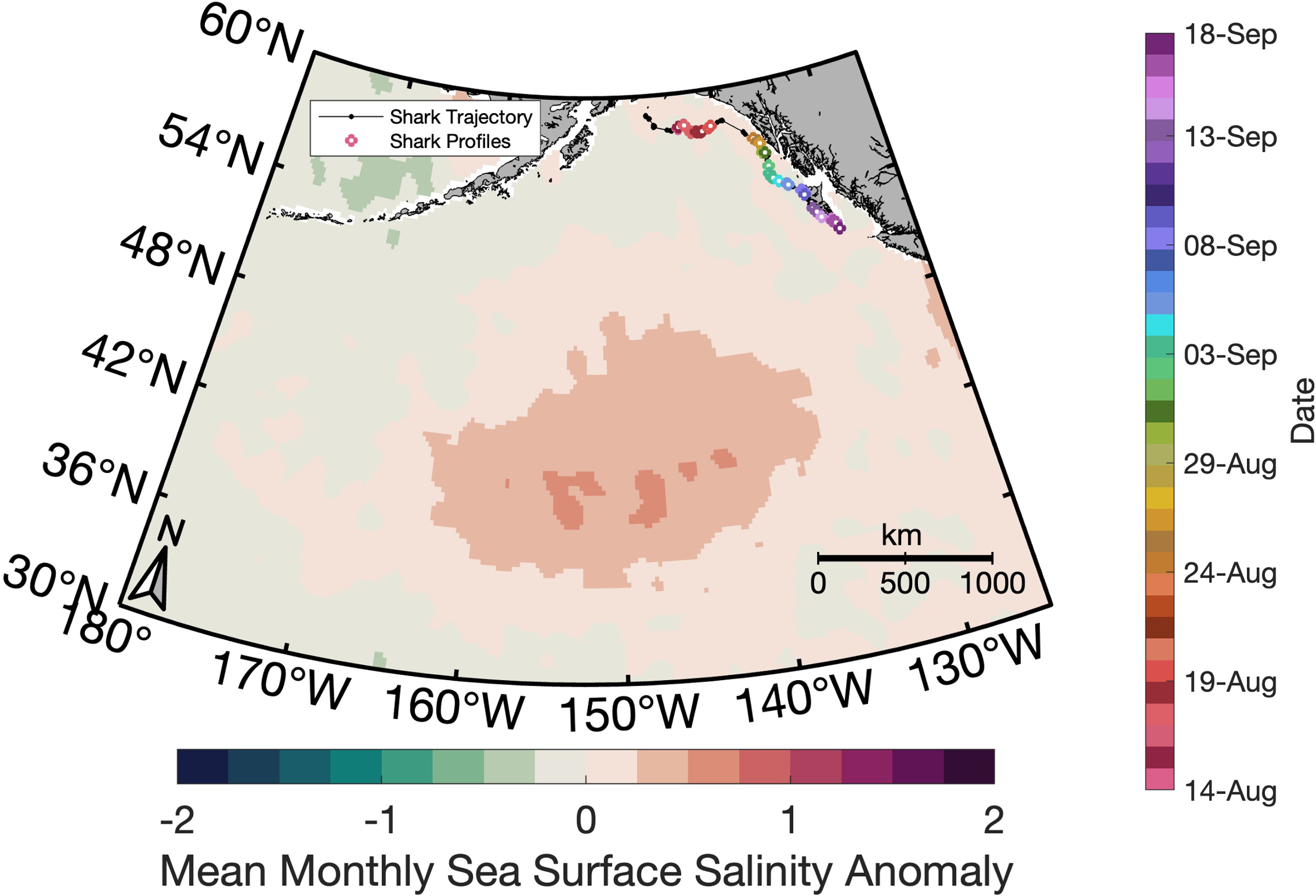


Fig. S7.

Mean sea surface salinity anomaly in the Gulf of Alaska during September 2015. Trajectory of the salmon shark equipped with the CTD-SRDL fin tag from ARGOS location estimates (black dots) with locations of temperature-salinity profiles (circles colored by date) is also showed.


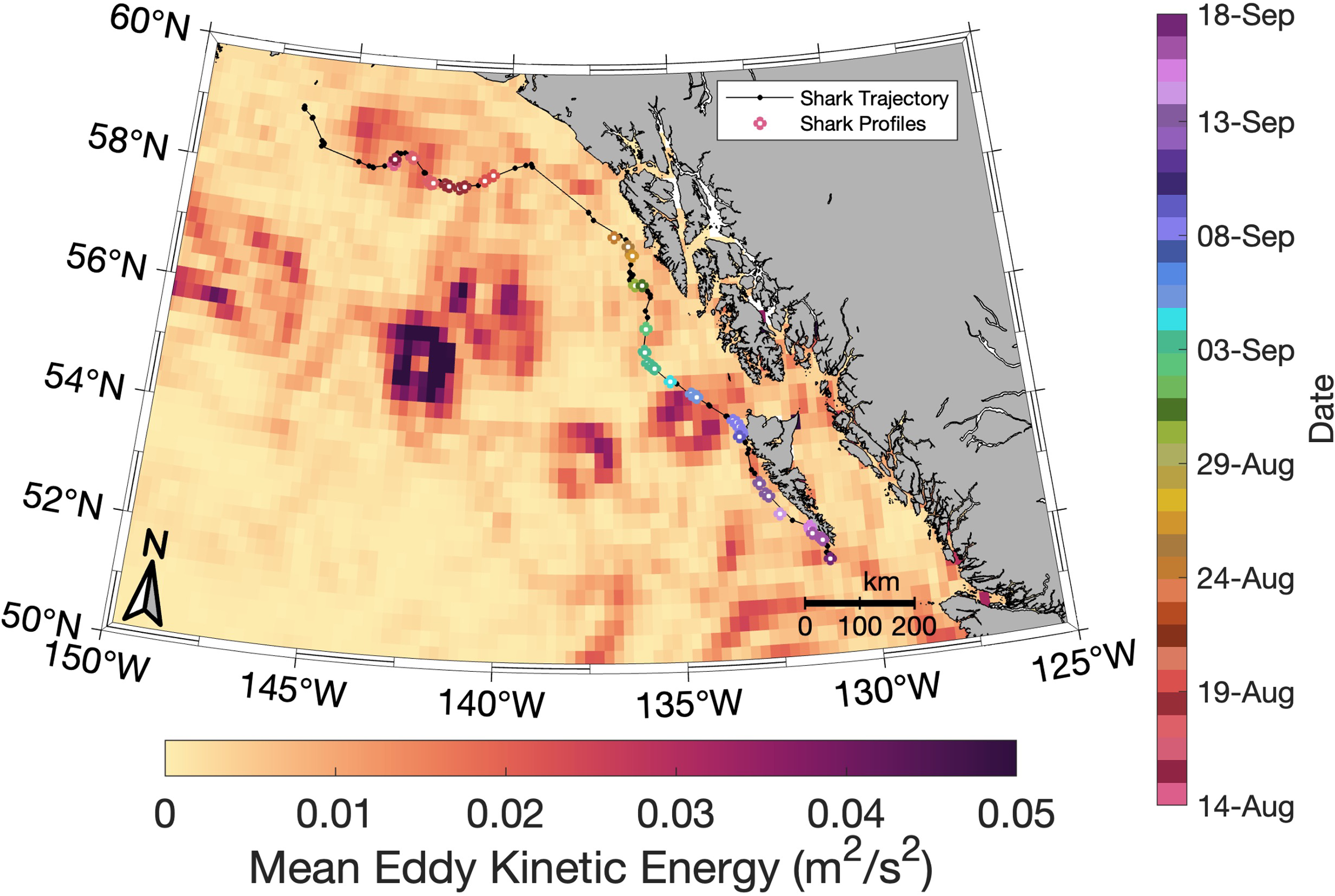


Fig. S8.

Mean eddy kinetic energy in the Gulf of Alaska between August 14 to September 18, 2015. Trajectory of the salmon shark equipped with the CTD-SRDL fin tag from ARGOS location estimates (black dots) with locations of temperature-salinity profiles (circles colored by date) is also showed.


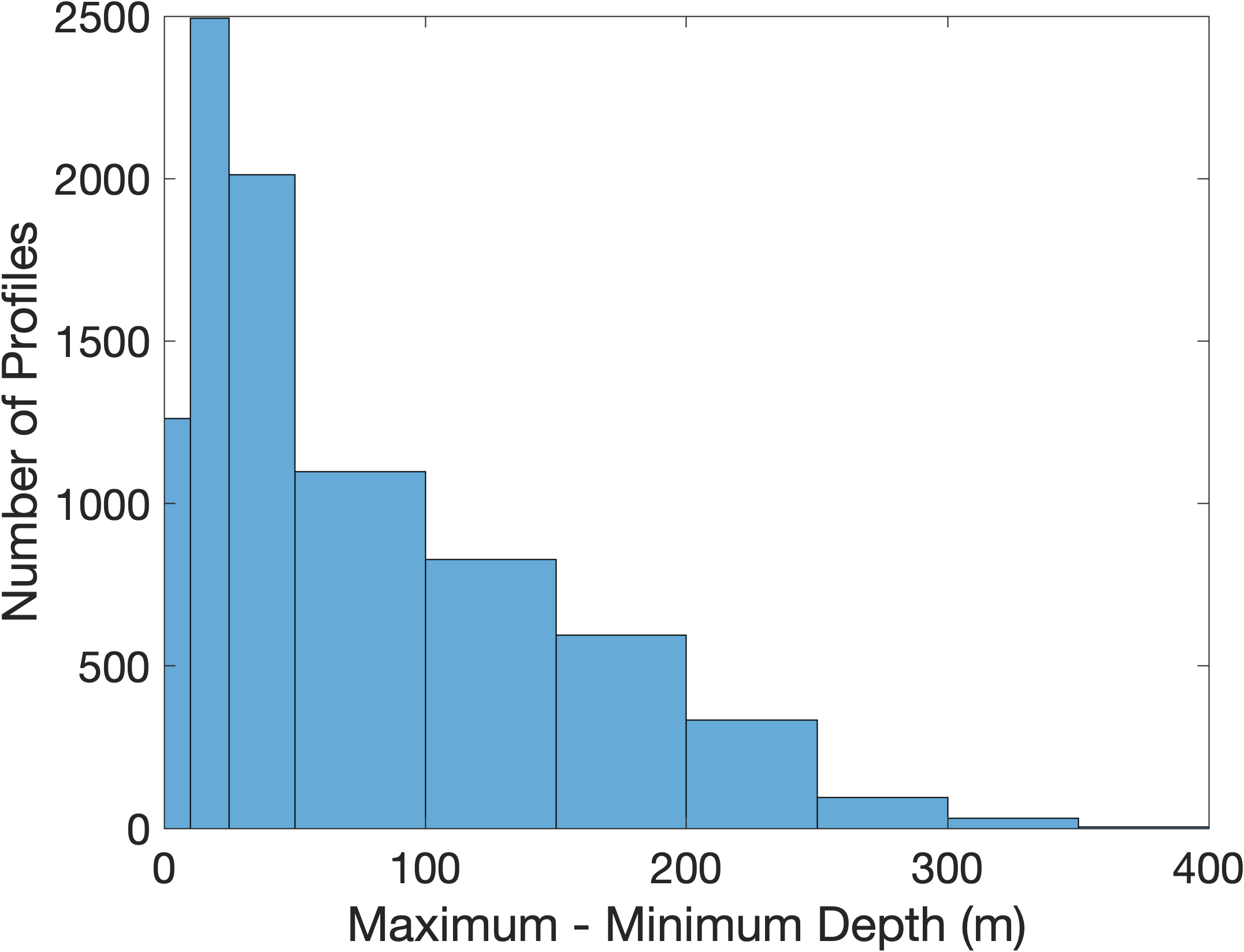


Fig. S9.

Distribution of depths spanned (i.e., maximum - minimum depth; m) by all temperature-depth profiles within the Gulf of Alaska extracted from 10 recovered PAT tags.


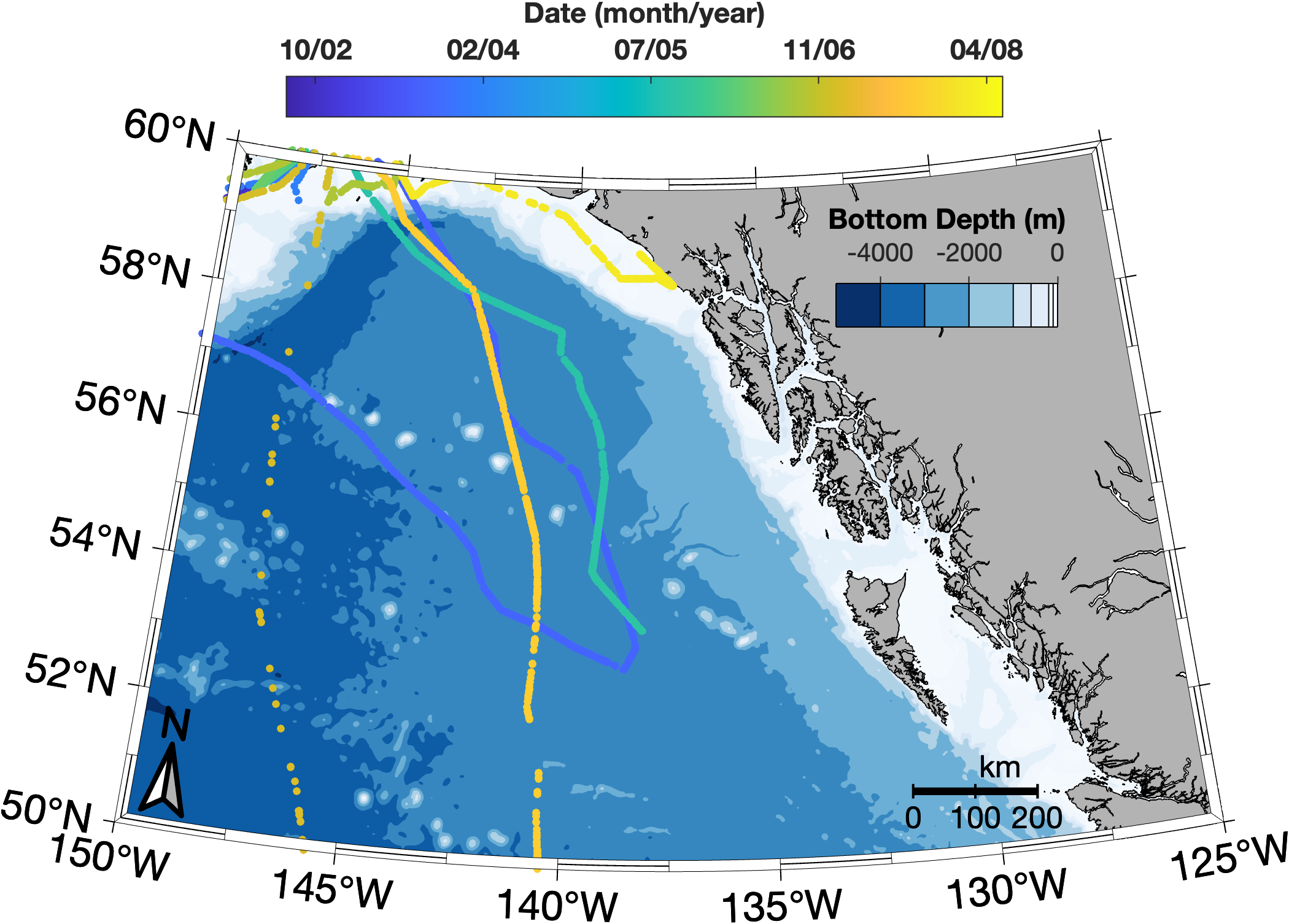


Fig. S10.

Location of temperature-depth profiles extracted from 10 recovered PAT tags in the Gulf of Alaska. Circles are colored by date on which the profile was recorded.


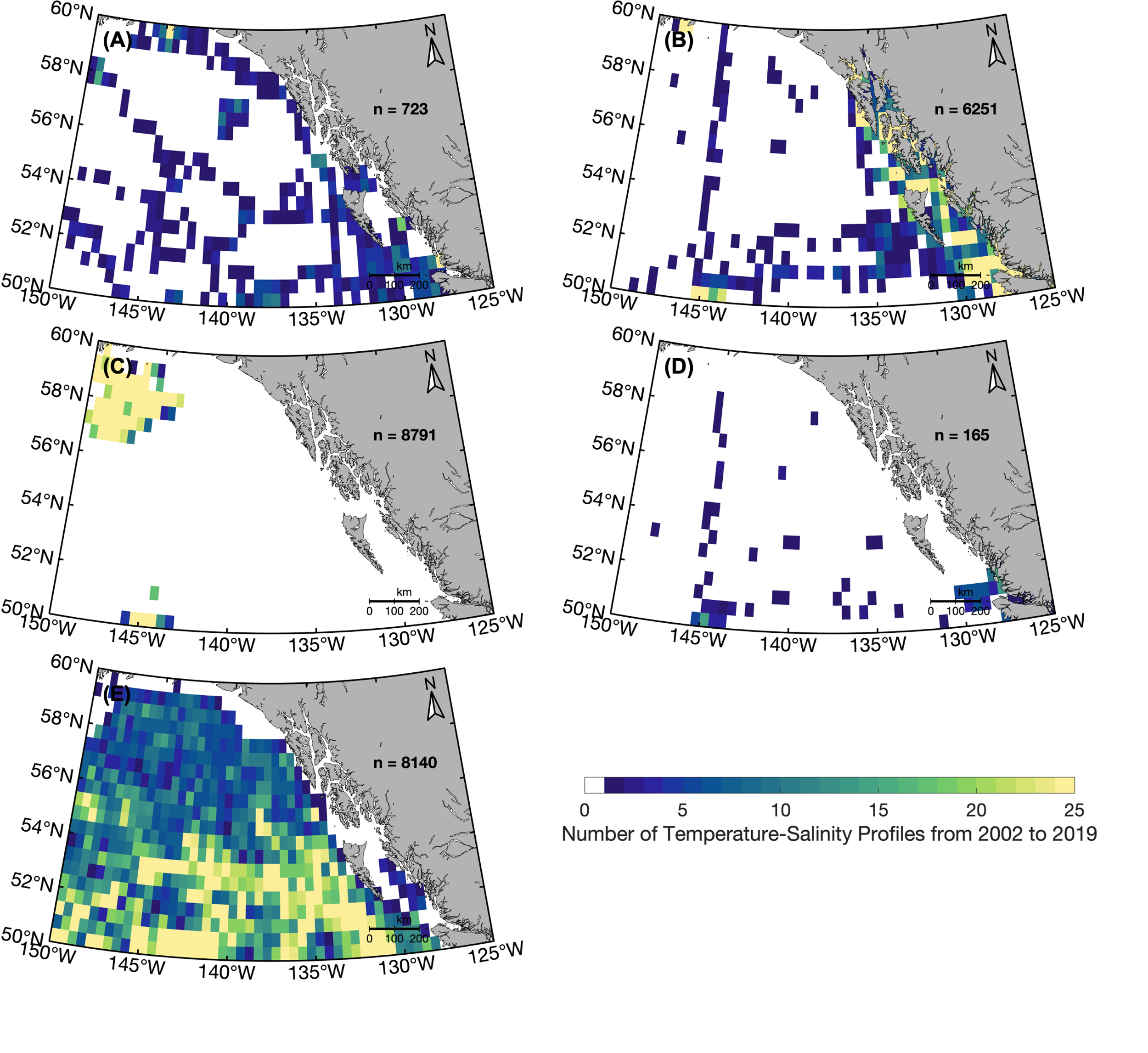


Fig. S11.

Number of temperature-salinity profiles from the World Ocean Database on days when salmon sharks were present between 2002 and 2019 in the Gulf of Alaska collected by (A) pinnipeds, (B) CTDs, (C) gliders, (D) ocean station data, and (E) Argo profiling floats. Bins are 0.5° latitude and 0.5° longitude.


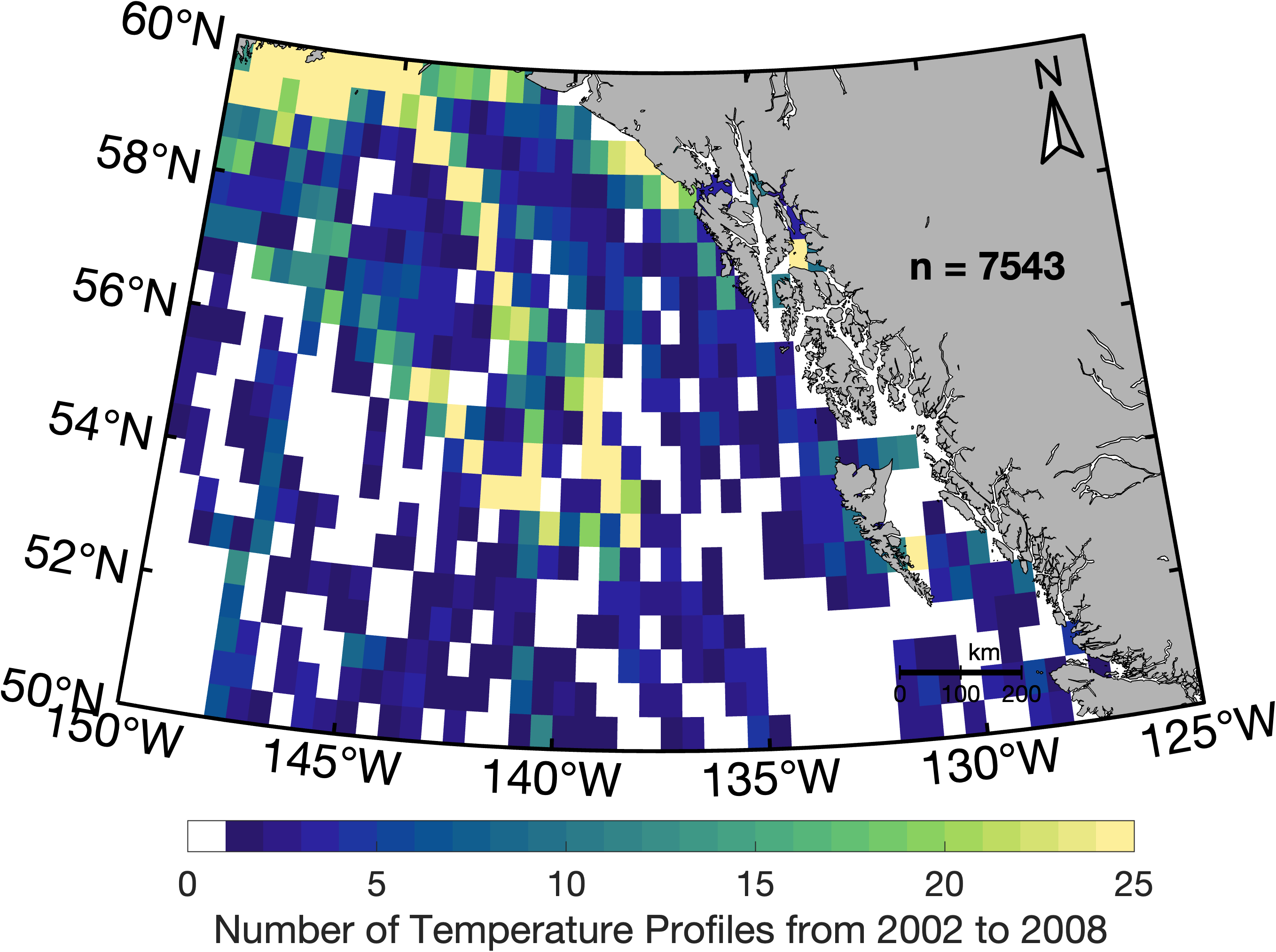


Fig. S12.

Number of shark-collected temperature-depth profiles (i.e., both PDTs and from recovered PAT tags) between 2002 and 2008 in the Gulf of Alaska. Bins are 0.5° latitude and 0.5° longitude.


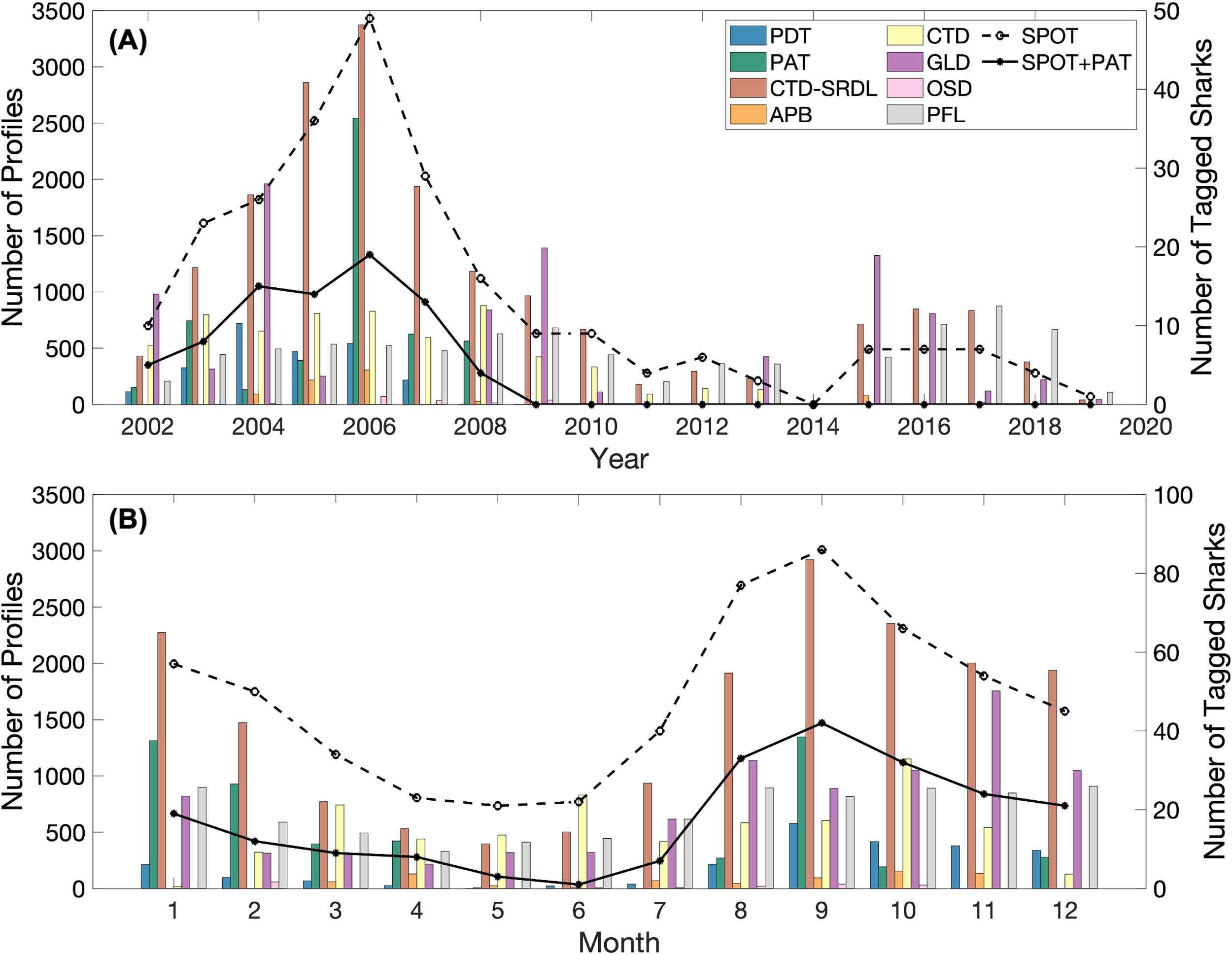


Fig. S13.

Minimum number of temperature-salinity profiles that could have been collected if all SPOT-tagged salmon sharks were instrumented with CTD-SRDL fin tags (red) as well as the number of PDTs from transmitted PAT tags (blue) and number of temperature-depth profiles from recovered PAT tags (green) by (A) year and (B) month. Also shown are the number of temperature-salinity profiles in the World Ocean Database on days when salmon sharks were present in the Gulf of Alaska collected by pinnipeds (orange), CTDs (yellow), gliders (purple), ocean station data (pink) and Argo profiling floats (grey). Lines indicate the number of SPOT-tagged (dashed black line and circles) and number of “double tagged” (i.e., SPOT+PAT; solid black line and asterisks) sharks.

Table S1.

Fixed depth points transmitted depending on dive depth. Point in brackets is transmitted in separate ARGOS message with the broken-stick depths.

| **Pressure (dbar)** | **Fixed Depths Used** | | | | | | | |
| --- | --- | --- | --- | --- | --- | --- | --- | --- |
| 10 | X | X | X | X | X | X | X | X |
| 12 | X |  |  |  |  |  |  |  |
| 14 | X | X |  |  |  |  |  |  |
| 16 | X |  |  |  |  |  |  |  |
| 18 | X |  |  |  |  |  |  |  |
| 20 | X | X | X | X | X | X | X | X |
| 22 | (X) |  |  |  |  |  |  |  |
| 24 | X |  |  |  |  |  |  |  |
| 26 |  | X |  |  |  |  |  |  |
| 30 |  | X | X | X | X | (X) |  |  |
| 36 |  | (X) |  |  |  |  |  |  |
| 40 |  | X | X | X |  |  |  |  |
| 50 |  | X | X | X | X | X | X | X |
| 60 |  |  | X | (X) |  |  |  |  |
| 80 |  |  | (X) |  | (X) |  |  |  |
| 100 |  |  | X | X | X | X | X | X |
| 150 |  |  |  | X | X | X | (X) |  |
| 200 |  |  |  |  | X | X | X | X |
| 300 |  |  |  |  |  | X | X | X |
| 400 |  |  |  |  |  |  | X | (X) |
| 500 |  |  |  |  |  |  |  | X |

Table S2.

Metadata for deployment of the CTD-SRDL fin tag on a female salmon shark. PCL = precaudal length.

| **Tag #** | **Tagging Date** | **PCL (cm)** | **Deployment Longitude (˚W)** | **Deployment Latitude (˚N)** | **Number of Geolocated CTD Profiles** | **Tag Design** |
| --- | --- | --- | --- | --- | --- | --- |
| 13219 | 8/5/2015 | 157 | 146.0544 | 60.76855 | 56 | Twin |

Table S3.

Descriptive statistics of the distributions of conservative temperature (Θ) and absolute salinity (*S_A_*) anomalies from CTD-SRDL fin tag.

|  | **Θ_anom_** | ***S_A_* _anom_** |
| --- | --- | --- |
| mean | 0.60 ºC | 0.07 g/kg |
| standard deviation | 1.15 ºC | 0.47 g/kg |
| skewness | -0.38 | 0.87 |
| kurtosis | 3.91 | 4.33 |
| minimum | -3.14 ºC | -0.86 g/kg |
| maximum | 4.20 ºC | 1.95 g/kg |

Table S4.

Eddies encountered by the salmon shark equipped with the CTD-SRDL fin tag along its trajectory.

| **Eddy Type** | **General Locale** | **Average Eddy Center Longitude (˚W)** | **Average Eddy Center Latitude (˚N)** | **# of CTD Profiles** | **Entrance Date & Time** | **Exit Date & Time** | **Total Time in Eddy (hours)** |
| --- | --- | --- | --- | --- | --- | --- | --- |
| ACE | near apex of GoA | 143° 19' 16.9" | 58° 22' 29.6" | 6 | 8/14/15 22:52 | 8/18/15 3:19 | 76.4 |
| CE | near apex of GoA | 141° 37' 30.9" | 57° 53' 39.1" | 3 | 8/19/15 6:25 | 8/19/15 18:51 | 12.4 |
| ACE | near apex of GoA | 140° 08' 12.0" | 58° 21' 43.3" | 0 | 8/21/15 5:02 | 8/21/15 5:24 | 0.4 |
| ACE | south of Sitka, AK | 135° 56' 13.4" | 55° 47' 01.8" | 2 | 9/2/15 23:39 | 9/3/15 13:07 | 13.5 |
| CE | south of Sitka, AK | 135° 38' 30.4" | 55° 00' 01.8" | 6 | 9/4/15 5:30 | 9/5/15 18:35 | 37.1 |
| ACE | south of Sitka, AK | 134° 54' 02.9" | 53° 52' 31.1" | 3 | 9/6/15 18:16 | 9/7/15 19:20 | 25.1 |
| CE | near Kunghit Island, BC | 131° 34' 47.0" | 51° 52' 31.9" | 3 | 9/17/15 16:00 | 9/17/15 20:47 | 4.8 |

Table S5.

All salmon sharks tagged with a SPOT tag in Port Gravina, Prince William Sound, Alaska between 2002 and 2015. Some sharks were “double tagged” with a SPOT and PAT tag. PCL = precaudal length.

See additional supplemental file named Table_S5.xlsx.

Table S6.

Female salmon sharks “double tagged” with a SPOT and PAT tag in Port Gravina, Prince William Sound, Alaska between 2002 and 2007 whose PAT tags were recovered. PCL = precaudal length.

| **TOPP ID** | **Tagging Date** | **Tagging Latitude (°N)** | **Tagging Longitude (°W)** | **Tag Models** | **PCL (cm)** | **Pop-Up Date** | **Pop-Up Latitude (°N)** | **Pop-Up Longitude (°W)** | **Days at Liberty** |
| --- | --- | --- | --- | --- | --- | --- | --- | --- | --- |
| 1702003 | 7/15/02 | 60.76 | -146.07 | PAT2, SPOT2 | 208 | 11/14/02 | 54.98 | -160.51 | 122 |
| 1703010 | 8/18/03 | 60.73 | -146.09 | PAT3, SPOT3 | 208 | 2/20/04 | 58.47 | -147.13 | 186 |
| 1704004 | 7/11/04 | 60.71 | -146.14 | PAT4, SPOT4 | 218 | 8/15/04 | 64.82 | -147.65 | 28 |
| 1704006 | 7/12/04 | 60.72 | -146.17 | PAT4, SPOT4 | 214 | 8/8/04 | 60.34 | -146.72 | 28 |
| 1704016 | 7/13/04 | 60.72 | -146.18 | PAT4, SPOT4 | 207 | 8/28/04 | 60.72 | -146.07 | 47 |
| 1705007 | 8/21/05 | 60.73 | -146.10 | PAT4, SPOT5 | 217 | 9/21/05 | 53.29 | -137.87 | 31 |
| 1705014 | 8/22/05 | 60.73 | -146.09 | PAT4, SPOT5 | 206 | 5/21/06 | 60.72 | -147.53 | 272 |
| 1706001 | 8/20/06 | 60.72 | -146.10 | PAT-MK10, SPOT5 | 212 | 5/16/07 | 45.80 | -142.53 | 269 |
| 1707010 | 8/23/07 | 60.73 | -146.09 | PAT-MK10, SPOT5 | 217 | 5/19/08 | 35.59 | -121.20 | 270 |
| 1707011 | 8/23/07 | 60.73 | -146.09 | PAT-MK10, SPOT5 | 192 | 2/19/08 | 59.70 | -139.49 | 180 |
| 1707016 | 8/24/07 | 60.73 | -146.09 | PAT-MK10, SPOT5 | 195 | 4/20/08 | 61.07 | -148.13 | 240 |
