# Supplementary material for: Novel CTD tag establishes shark fins as ocean observing platforms
Source: Sci Rep. 2024 Jun 15;14:13837. doi: 10.1038/s41598-024-63543-5 (PMC11180183; doi:10.1038/s41598-024-63543-5)
Supplement: Supplementary file 2 — Supplementary Table S5. [file 41598_2024_63543_MOESM2_ESM.pdf]

	A	B	C	D	E	F	G	H	I	J	K
1	**TOPP ID**	**Tagging Date**	**Tagging Latitude**	**Tagging Longitude**	**PAT Tag Model**	**SPOT Tag Model**	**PCL (cm)**	**Sex**	**PAT Pop-Up Date**	**PAT Pop-Up Latitude**	**PAT Pop-Up Longitude**
2	1702001	7/14/02	60.76	-146.07	PAT2	SPOT2	207	F	11/14/02	41.65	-124.92
3	1702002	7/14/02	60.76	-146.06	PAT2	SPOT2	196	F	11/14/02	44.88	-124.37
4	1702003	7/15/02	60.76	-146.07	PAT2	SPOT2	208	F	11/14/02	54.98	-160.51
5	1702004	7/15/02	60.76	-146.07	PAT2	SPOT2	216	F	1/13/03	60.78	-146.69
6	1702005	7/16/02	60.71	-146.18	PAT2	SPOT2	218	F	1/13/03	57.32	-133.67
7	1702006	7/16/02	60.71	-146.18		SPOT2	212	F			
8	1702008	7/17/02	60.72	-146.18	PAT2	SPOT2	218	F			
9	1702009	7/17/02	60.72	-146.16		SPOT2	214	F			
10	1702010	7/17/02	60.72	-146.18		SPOT2	201	F			
11	1702011	7/17/02	60.71	-146.19		SPOT2	226	F			
12	1703001	8/15/03	60.73	-146.09	PAT3	SPOT2	216	F	2/16/04	35.48	-135.79
13	1703002	8/15/03	60.72	-146.10	PAT3	SPOT2	201	F	11/20/03	28.62	-149.18
14	1703004	8/16/03	60.73	-146.08		SPOT2	218	F			
15	1703005	8/17/03	60.71	-146.09		SPOT2	213	F			
16	1703006	8/17/03	60.73	-146.09	PAT3	SPOT2	220	F	5/20/04	59.60	-144.65
17	1703007	8/17/03	60.73	-146.08	PAT3	SPOT2		F			
18	1703008	8/17/03	60.73	-146.09		SPOT3	205	F			
19	1703009	8/18/03	60.73	-146.09	PAT3	SPOT3	219	F	2/20/04	22.27	-160.24
20	1703010	8/18/03	60.73	-146.09	PAT3	SPOT3	208	F	2/20/04	58.47	-147.13
21	1703011	8/18/03	60.73	-146.09	PAT3	SPOT3	216	F	2/20/04	43.04	-134.24
22	1703012	8/18/03	60.73	-146.09		SPOT2	209	F			
23	1703013	8/19/03	60.73	-146.09	PAT3	SPOT3	218	F	2/20/04	31.53	-142.76
24	1703014	8/19/03	60.73	-146.09		SPOT3	229	F			
25	1703015	8/19/03	60.73	-146.09		SPOT3	229	F			
26	1703016	8/19/03	60.73	-146.09	PAT3	SPOT3	217	F			
27	1704001	7/11/04	60.71	-146.13	PAT4	SPOT4	219	F	4/10/05	42.45	-124.75

	A	B	C	D	E	F	G	H	I	J	K
28	1704002	7/11/04	60.71	-146.15	PAT4	SPOT4	222	F	4/11/05	33.96	-120.81
29	1704003	7/11/04	60.71	-146.13	PAT4	SPOT4	212	F	11/9/04	59.94	-148.00
30	1704004	7/11/04	60.71	-146.14	PAT4	SPOT4	218	F	8/15/04	64.82	-147.65
31	1704005	7/12/04	60.69	-146.19	PAT4	SPOT4	188	F	1/8/05	57.17	-152.83
32	1704006	7/12/04	60.72	-146.17	PAT4	SPOT4	214	F			
33	1704007	7/12/04	60.71	-146.17	PAT4	SPOT4	221	F	4/12/05	40.91	-144.83
34	1704008	7/12/04	60.72	-146.17		SPOT4	224	F			
35	1704009	7/12/04	60.72	-146.18	PAT4	SPOT4	214	F	4/9/05	60.60	-147.29
36	1704011	7/13/04	60.72	-146.18	PAT4	SPOT4	215	F	1/9/05	56.90	-153.10
37	1704013	7/13/04	60.72	-146.18	PAT4	SPOT4	224	F	1/9/05	59.29	-147.11
38	1704014	7/13/04	60.72	-146.18	PAT4	SPOT4	202	F	1/9/05	34.18	-123.34
39	1704015	7/13/04	60.72	-146.18	PAT4	SPOT4	216	F			
40	1704017	7/13/04	60.72	-146.18	PAT4	SPOT4	223	F	1/9/05	57.90	-154.13
41	1704018	7/13/04	60.72	-146.18	PAT4	SPOT4	211	F			
42	1704019	7/14/04	60.72	-146.18	PAT4	SPOT4	229	F			
43	1704020	7/14/04	60.71	-146.18	PAT4	SPOT4	218	F	3/12/05	30.12	-134.62
44	1704021	7/15/04	60.72	-146.17	PAT4	SPOT4	212	F			
45	1705001	8/20/05	60.73	-146.09	PAT4	SPOT5	220	F	8/6/06	60.66	-146.32
46	1705002	8/21/05	60.73	-146.09	PAT4	SPOT5	191	F	5/21/06	60.32	-146.61
47	1705003	8/21/05	60.73	-146.09	PAT4	SPOT5	208	F	8/22/06	54.07	-133.17
48	1705005	8/21/05	60.73	-146.09	PAT4	SPOT5	209	F	5/21/06	60.12	-146.65
49	1705007	8/21/05	60.73	-146.10	PAT4	SPOT5	217	F	9/21/05	53.29	-137.87
50	1705008	8/21/05	60.73	-146.09	PAT4	SPOT5	211	F	4/23/06	37.86	-123.38
51	1705011	8/22/05	60.73	-146.09	PAT4	SPOT5	198	F	5/21/06	60.31	-146.63
52	1705012	8/22/05	60.73	-146.08	PAT4	SPOT5	198	F			
53	1705013	8/22/05	60.73	-146.09	PAT4	SPOT5	210	F	5/2/06	36.53	-138.60
54	1705014	8/22/05	60.73	-146.09	PAT4	SPOT5	206	F	5/21/06	60.72	-147.53
55	1705015	8/22/05	60.73	-146.09	PAT4	SPOT5	202	F			

	A	B	C	D	E	F	G	H	I	J	K
56	1705016	8/22/05	60.73	-146.09	PAT4	SPOT5	200	F	5/21/06	34.01	-121.11
57	1705018	8/23/05	60.73	-146.09	PAT4	SPOT5	211	F	9/28/05	52.88	-132.32
58	1705019	8/23/05	60.73	-146.10		SPOT5	205	F			
59	1705020	8/23/05	60.73	-146.09	PAT4	SPOT5	209	F			
60	1705021	8/24/05	60.73	-146.11	PAT4	SPOT5	190	F	11/20/05	56.92	-137.18
61	1705022	8/24/05	60.73	-146.09		SPOT5	191	F			
62	1705024	8/24/05	60.73	-146.09		SPOT5	208	F			
63	1705025	8/24/05	60.73	-146.08	PAT4	SPOT5	209	F			
64	1705026	8/24/05	60.73	-146.10		SPOT5	212	F			
65	1705027	8/24/05	60.73	-146.09		SPOT5	214	F			
66	1706001	8/20/06	60.72	-146.10	PAT-MK10	SPOT5	212	F	5/16/07	45.80	-142.53
67	1706004	8/20/06	60.39	-146.10	PAT-MK10	SPOT5	192	F			
68	1706005	8/21/06	60.72	-146.10	PAT-MK10	SPOT5	233	F			
69	1706006	8/21/06	60.72	-146.11	PAT-MK10	SPOT5	199	F			
70	1706008	8/21/06	60.72	-146.10	PAT-MK10	SPOT5	227	F			
71	1706009	8/21/06	60.72	-146.10	PAT-MK10	SPOT5	206	F			
72	1706010	8/21/06	60.72	-146.10	PAT-MK10	SPOT5	211	F			
73	1706011	8/21/06	60.72	-146.10	PAT-MK10	SPOT5	208	F	5/19/07	46.32	-137.52
74	1706012	8/22/06	60.72	-146.10	PAT-MK10	SPOT5	215	F			
75	1706014	8/22/06	60.72	-146.10	PAT-MK10	SPOT5	210	F	9/25/06	56.96	-137.72
76	1706016	8/22/06	60.72	-146.10	PAT-MK10	SPOT5	200	F	4/23/07	30.72	-118.58
77	1706017	8/22/06	60.72	-146.11	PAT-MK10	SPOT5	204	F	2/14/07	39.53	-135.09
78	1706018	8/22/06	60.72	-146.10	PAT-MK10	SPOT5	218	F			
79	1706022	8/23/06	60.72	-146.10	PAT-MK10	SPOT5	219	F	10/24/06	56.18	-151.25
80	1706023	8/23/06	60.72	-146.11	PAT-MK10	SPOT5	197	F	4/20/07	30.59	-118.14
81	1706026	8/24/06	60.72	-146.11	PAT-MK10	SPOT5	198	F	5/21/07	32.18	-120.79
82	1706027	8/24/06	60.72	-146.11		miniSPOT5	214	F			
83	1706029	8/24/06	60.72	-146.10	PAT-MK10	SPOT5	214	F	3/22/07	32.93	-137.55

	A	B	C	D	E	F	G	H	I	J	K
84	1706031	8/24/06				miniSPOT5	191	F			
85	1706034	8/25/06	60.72	-146.10	PAT-MK10	SPOT5	241	F	9/26/06	46.15	-132.27
86	1706036	8/25/06	60.72	-146.10	PAT-MK10	SPOT4	216	F	2/21/07	43.36	-144.27
87	1706037	8/25/06	60.72	-146.10	PAT-MK10	SPOT4	213	F	2/21/07	38.08	-125.63
88	1706039	8/26/06	60.72	-146.10		miniSPOT5	215	U			
89	1706041	8/26/06	60.72	-146.10	PAT-MK10	SPOT4	198	F	4/23/07	37.05	-122.75
90	1707001	8/21/07	60.73	-146.08	PAT-MK10	SPOT5	195	F	12/17/07	53.87	-160.95
91	1707003	8/21/07	60.73	-146.09		SPOT5	198	F			
92	1707004	8/22/07	60.73	-146.09	PAT-MK10	SPOT5	208	F	9/20/07	60.73	-146.12
93	1707005	8/22/07	60.73	-146.09		SPOT5	208	F			
94	1707006	8/22/07	60.73	-146.09		SPOT5	197	F			
95	1707008	8/22/07	60.73	-146.09	PAT-MK10	SPOT5	219	F	2/18/08	31.83	-120.78
96	1707009	8/23/07	60.76	-146.08		SPOT5	203	F			
97	1707010	8/23/07	60.73	-146.09	PAT-MK10	SPOT5	217	F	5/19/08	35.59	-121.20
98	1707011	8/23/07	60.73	-146.09	PAT-MK10	SPOT5	192	F	2/19/08	59.70	-139.49
99	1707012	8/23/07	60.73	-146.09		SPOT5	219	F			
100	1707013	8/23/07	60.73	-146.09		SPOT5	199	F			
101	1707014	8/23/07	60.73	-146.09	PAT-MK10	SPOT5	218	F	12/5/07	60.82	-147.22
102	1707015	8/24/07	60.73	-146.09	PAT-MK10	SPOT5	200	F	1/5/08	59.90	-148.06
103	1707016	8/24/07	60.73	-146.09	PAT-MK10	SPOT5	195	F	4/20/08	61.07	-148.13
104	1707017	8/24/07	60.73	-146.09	PAT-MK10	SPOT5	204	F	3/4/08	60.75	-146.93
105	1707018	8/24/07	60.73	-146.08	PAT-MK10	SPOT5	214	F			
106	1708001	8/18/08	60.72	-146.11		SPOT5	176	F			
107	1708003	8/20/08	60.71	-146.11		SPOT5	190	F			
108	1710012	7/18/10	60.77	-146.05		SPOT5	185	F			
109	1710014	7/18/10	60.77	-146.05		SPOT5	202	F			
110	1710019	7/19/10	60.77	-146.05		SPOT5	188	F			
111	1710020	7/19/10	60.77	-146.05		SPOT5	175	F			

	A	B	C	D	E	F	G	H	I	J	K
112	1710021	7/19/10	60.77	-146.05		SPOT5	186	F			
113	1710022	7/19/10	60.77	-146.05		SPOT5	188	F			
114	1710023	7/19/10	60.77	-146.05		SPOT5	163	F			
115	1712001	7/19/12	60.77	-146.05		SPOT5	193	F			
116	1712002	7/20/12	60.77	-146.06		SPOT5	182	F			
117	1712003	7/20/12	60.77	-146.06		SPOT5	189	F			
118	1715001	8/6/15	60.77	-146.06		SPOT5	151	F			
119	1715002	8/5/15	60.77	-146.05		SPOT5	166	F			
120	1715003	8/6/15	60.77	-146.05		SPOT5	168	F			
121	1715004	8/6/15	60.77	-146.06		SPOT5	172	F			
122	1715005	8/6/15	60.77	-146.05		SPOT5	201	F			
123	1715005	8/6/15	60.77	-146.05		SPOT5	201	F			
124	1715006	8/5/15	60.77	-146.05		SPOT5	183	F			
125	1715008	8/6/15	60.77	-146.05		SPOT5	196	F			
126	1715010	8/6/15	60.77	-146.05		SPOT5	185	F			
